# Schoolgirls’ experience and appraisal of menstrual absorbents in rural Uganda: a cross-sectional evaluation of reusable sanitary pads

**DOI:** 10.1186/s12978-016-0260-7

**Published:** 2016-12-07

**Authors:** Julie Hennegan, Catherine Dolan, Maryalice Wu, Linda Scott, Paul Montgomery

**Affiliations:** 1 Centre for Evidence-Based Intervention, University of Oxford, Oxford, UK; 2 SOAS, University of London, London, UK; 3 Applied Technologies for Learning in the Arts and Sciences, University of Illinois, Urbana-Champaign, USA; 4 Said Business School, University of Oxford, Oxford, UK

**Keywords:** Menstrual hygiene management, Adolescent girls, Education, Sanitary products, Menstrual health, Reproductive health

## Abstract

**Background:**

Governments, multinational organisations, and charities have commenced the distribution of sanitary products to address current deficits in girls’ menstrual management. The few effectiveness studies conducted have focused on health and education outcomes but have failed to provide quantitative assessment of girls’ preferences, experiences of absorbents, and comfort. Objectives of the study were, first, to quantitatively describe girls’ experiences with, and ratings of reliability and acceptability of different menstrual absorbents. Second, to compare ratings of freely-provided reusable pads (AFRIpads) to other existing methods of menstrual management. Finally, to assess differences in self-reported freedom of activity during menses according to menstrual absorbent.

**Methods:**

Cross-sectional, secondary analysis of data from the final survey of a controlled trial of reusable sanitary padand puberty education provision was undertaken. Participants were 205 menstruating schoolgirls from eight schools in rural Uganda. 72 girls who reported using the intervention-provided reusable pads were compared to those using existing improvised methods (predominately new or old cloth).

**Results:**

Schoolgirls using reusable pads provided significantly higher ratings of perceived absorbent reliability across activities, less difficulties changing absorbents, and less disgust with cleaning absorbents. There were no significant differences in reports of outside garment soiling (OR 1.00 95%CI 0.51–1.99), or odour (0.84 95%CI 0.40–1.74) during the last menstrual period. When girls were asked if menstruation caused them to miss daily activities there were no differences between those using reusable pads and those using other existing methods. However, when asked about activities avoided during menstruation, those using reusable pads participated less in physical sports, working in the field, fetching water, and cooking.

**Conclusions:**

Reusable pads were rated favourably. This translated into some benefits for self-reported involvement in daily activities, although reports of actual soiling and missing activities due to menstruation did not differ. More research is needed comparing the impact of menstrual absorbents on girls’ daily activities, and validating outcome measures for menstrual management research.

**Electronic supplementary material:**

The online version of this article (doi:10.1186/s12978-016-0260-7) contains supplementary material, which is available to authorized users.

## Plain language summary

Governments and charity organisations have proposed providing resources, such as reusable pads, to address the barrier that managing menstruation may present to girls’ dignity, health, education, and psychosocial wellbeing. However, studies of sanitary product provision to date have not quantitatively tested girls’ perceptions and experience of such products.

This study describes and compares schoolgirls’ experiences of different menstrual absorbents. The work provides a follow-up to a controlled trial of reusable sanitary pad and puberty education provision which tested improvements in school attendance. By comparing the experiences between girls who received reusable sanitary pads as part of the trial and girls using other existing methods of menstrual absorption (predominantly cloth), this study provides insight into girls’ comfort and preferences. A sample of 205 schoolgirls, from eight schools in rural Uganda were included in the study. 72 girls who were using the reusable pads provided in the intervention study were compared with those using existing improvised methods. A few girls were using disposable sanitary pads, and their results are displayed separately. Results found that girls rated the reusable pads positively. Girls using the reusable pads were more likely to report they were easy to change, and were not disgusting to clean. Those using the reusable pads rated them as more reliable to keep them from soiling during a range of activities. Despite more positive perceptions, there were no differences in girls’ reports of soiling outer garments or experiencing odour during the last menstrual period. Results of the study are important in understanding girls’ preferences and experiences of menstrual absorbents in low income contexts.

## Background

Menstruation is a healthy and natural process for women and adolescent girls. The management of menstruation has varied over time and differs with available resources, cultural traditions, and preferences. There is increasing recognition that in low-income contexts, women struggle to manage menstruation in effective and hygienic ways [1]. This has been linked to negative impacts on health, education and wellbeing [2, 3]. A lack of clean and reliable menstrual absorbents has received much of the attention, with interventions focussed on the provision of these products to improve poor menstrual hygiene and associated consequences [4, 5]. There has been a proliferation of programs and organisations providing girls with menstrual absorbents, most frequently reusable or disposable pads [6–8]. A recent systematic review of studies evaluating the effectiveness of such interventions found few rigorous trials had been conducted [5]. The review identified only 3 studies of menstrual product provision, each assessing a different product; menstrual cups, [9] disposable sanitary pads, [10] and home-made reusable pads [11].

Whilst results of these trials seem promising with regard to school attendance, some have criticised menstrual management research for taking for granted the inadequacy of existing local methods [12]. Further, focus on hygiene and education outcomes may have minimised the importance of women and girls’ right to dignity and comfort during menstruation [2, 13]. Trials and observational studies have often overlooked girls’ perceptions of absorbent acceptability, reliability and comfort, as well as absorbents’ impact on self-efficacy. These outcomes are critical for many reasons. Firstly, school absenteeism during menstruation is frequently attributed to soiling or the fear of garment soiling. As a result, trials providing menstrual absorbents may assume that observed improvements to school attendance result from a reduced risk of soiling, but none have reported on this outcome explicitly or tested it as a mediating pathway. Secondly, menstrual management programs typically provide only one type of product, which may be delivered alongside explicit education or proscriptive norms which discourage the use of existing improvised absorbents such as cloth. This could be seen as limiting girls’ choices for menstrual management, particularly in comparison to high-income contexts where women and girls may use a range of different products during menses. This places more pressure on ensuring the acceptability and adequacy of the products provided before interventions are implemented at scale. Further, if trials focus exclusively on school attendance and fail to report on other outcomes which have been neglected to date such as comfort, acceptability, or self-efficacy, sanitary product distribution interventions may be abandoned if education provision alone is found to be equally effective in improving attendance, as has been the case in recent studies [10, 14].

Girls’ dignity must be kept at the forefront of primary research and intervention studies of menstrual management. Assessment of reliability, comfort, and satisfaction with activities such as changing and washing absorbents put girls’ agency and preferences at the centre of program evaluation. The present study is the first to do this quantitatively, providing a cross-sectional follow-up to the *Menstruation and the Cycle of Poverty* trial [14] and evaluates reusable pads as an alternative to existing improvised methods. Reusable pads are assessed in terms of girls’ experiences, preferences and self-reported leaking, odour, and avoidance of daily activities during menses. The study compares ratings between those using reusable pads provided as part of the trial, and those using existing improvised menstrual absorbents (including cloth, toilet paper, and underwear alone). A small group of girls reported using commercially produced sanitary pads and their ratings are displayed separately.

Past trials of menstrual product provision have addressed some of these questions with qualitative reports or field observations of product acceptability and uptake [9, 11, 15]. The *Menstruation and the Cycle of Poverty* trial [14] assessed here, conducted a pilot assessment of menstrual products. Prior to trial implementation girls in a different region provided feedback on proposed absorbents for intervention compared to each other and existing methods such as cloth or underwear alone (see [16]). While reported reusable pad acceptability was high, only 44% of the sample reported they would switch from their existing methods (possibly due to a high baseline rate of disposable sanitary pad use). Other studies have found qualitative evidence of acceptability of menstrual cups, but it is unclear if these would be preferred to other methods if offered [17]. A recent study of women in a high income context, where other products such as pads and tampons are available, found only 55% reported they would continue using a menstrual cup after an acceptability study [18]. Whilst some positive endorsement is evidenced here, findings indicate individual differences in preferences, and that acceptability and experience measures must be taken into account in trials of product provision interventions beyond preliminary qualitative studies. Quantitatively reported outcomes would allow comparison between different products trialled. While some have suggested that large trials of multiple products may be needed to best maximise outcomes for women and girls, [4, 17] quantitative reports across studies of girls’ experiences would also allow for comparisons and help identify products which have a demonstrated benefit for health and education outcomes, as well as girls’ comfort. This evaluation is needed to provide agents in the field and policy makers with the breadth of information necessary for large-scale dissemination of programs to be considered.

### The present study

This paper follows-up the *Menstruation and the Cycle of Poverty* trial with a secondary analysis of data from the surveys conducted at the end of the trial. The trial and primary outcomes have been described elsewhere (see [14]). In brief, the study was conducted between January 2012 and December 2014 in Kamuli district, Uganda, in rural areas characterised by poor performance on health and education indicators, and a literacy rate below the national average [14, 19]. Eight schools were quasi-randomised (in alphabetical order) to one of four conditions: puberty education alone, reusable pad provision alone, puberty education and reusable pad provision, or no intervention control. The education provided was a 1.25 h session following the Straight Talk training guide (see [14]). While the pilot study in Ghana (see [10]) provided disposable pads for improved school attendance, the *Menstruating and the Cycle of Poverty* trial tested reusable pads (AFRIpads, described in methods). This change followed environmental concerns and difficulties disposing of single-use pads in the pilot, as well as the high cost and limited availability of disposable pads for long-term provision [16].

This paper presents a secondary analysis of the final survey data to extend upon the trial findings by providing a detailed assessment of girls’ perceptions of the pads provided, their comfort, as well as the impact of the reusable pads on daily activities beyond school attendance.

## Objectives

1. To describe girls’ access and use of existing menstrual absorbents in the survey sample.
2. To compare the experience, including the ease of changing and cleaning menstrual absorbents, between those using reusable pads and those using existing improvised methods.
3. To compare girls’ ratings of absorbent reliability in different situations between reusable pads and existing improvised methods.
4. To test if girls using reusable pads felt greater freedom to participate in daily activities than those using existing methods.
5. To provide a brief description of girls’ appraisals of the reusable pads including how much they liked them and if they would purchase the pads if not freely available.

## Methods

This study was conducted and reported according to best practice guidelines in the Strengthening the Reporting of Observational studies in Epidemiology statement [20]. The checklist for cross-sectional studies is reported in Additional file 1.

### Participants

Girls attending the trial schools at baseline were recruited into the *Menstruation and the Cycle of Poverty* trial. In addition, girls who transferred into the study schools during the trial were included in intervention delivery (of reusable pads and education) and in the final survey as not to stigmatise trial girls or discriminate against non-trial girls in resource provision. Similarly, non-menstruating girls were surveyed as not to identify menstruating girls. While those who transferred to the study schools could not be included in the intention-to-treat analyses of primary trial outcomes, they were incorporated in the final survey data set for secondary analyses.

Across the eight schools, 435 girls completed the final follow-up survey of the *Menstruation and The Cycle of Poverty* trial. Of these, 205 had reached menarche at the time of survey and were included in the present study.

In the present study, 145 girls (70.7%) were attending the trial schools at baseline and a part of the *Menstruating and the Cycle of Poverty* trial intention-to-treat sample. Of these, 67 (46.2%) received the reusable pads. A further 60 girls who had not been attending the study schools at baseline, but transferred into these schools during the trial are also included in the present study. Of these girls, 20 (33.3%) had received reusable sanitary pads.

### Materials

**Fig. 1:**
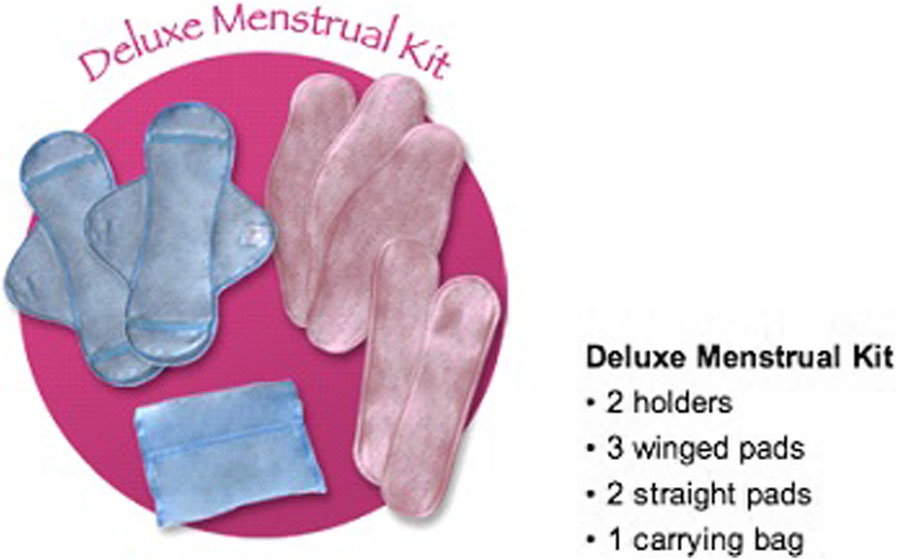
AFRIpads menstrual kits, 2012 (source: http://www.afripads.com/)

Reusable pads were provided to girls in four of the eight trial schools (for the reusable pads alone, and reusable pads and puberty education conditions). These were provided in October 2012, and March 2014. Girls were given a single pack of AFRIpads, underwear, and a small quantity of omo soap (1 sachet, 45 g). AFRIpads are a reusable cloth pad produced locally in Uganda. Packs provided included soil-resistant ‘base’ pads that fastened to underwear, 3 winged pads, and 3 straight pads, along with 2 bags for transporting pads (see http://www.afripads.com/). This was considered the ‘deluxe menstrual kit’ by AFRIpads at the time of delivery (see Fig. 1). Instructions on using and cleaning the reusable pads were provided by trained local research assistants alongside delivery.

### Measures

The follow-up survey was administered in November 2014. English surveys were loaded on iPads (using an offline app for *SurveyGizmo*) and delivered verbally in the local language (Lusoga), with trained research assistants from the partner NGO inputting responses in English. Research assistants were local young women, and visited the study schools in teams. For the survey, girls were taken aside to a private space and interviewed individually. If girls were absent on the day of interview, additional school sweeps were undertaken to survey all girls. Survey items required a response before progressing, or interviewers could indicate a ‘d*on’t know/no answer’* response if girls’ did not provide an answer. Survey interviews lasted approximately 30–40 min for menstruating girls.

The survey was developed by the research team in consultation with stakeholders and the partner NGO. Core items had been piloted in Ghana [10, 15]. Qualitative feasibility and acceptability work was undertaken prior to trial implementation, both in the local area, and similar regions (see [16]). This further shaped question development, as did existing menstrual hygiene literature. For transparency all survey items are reported in full in Additional file 2.

**Participant characteristics.** Girls self-reported their age, school grade, and how long it took them to get to school each day.

**Menstrual absorbent use.** Menstrual absorbent use was captured through the item: “*What do you usually use to catch/absorb your MP (menstrual period)?”* and girls provided a free response and resulted in the following responses: AFRIpad, cloth, sanitary pad or other items including toilet paper, underwear alone, mattress, or sponge. Follow-up items asked girls who provided their menstrual absorbent as a free response, responses included: the girls’ mother, another family member, or herself. Girls were asked where absorbents were obtained which was used to code if new or old cloth was being used. The menstrual absorbent categories are the same as reported in an additional secondary analysis paper on menstrual hygiene practices [21].

Girls provided number responses to questions: *“how many pieces of cloth do you have?”* and *“how many pieces of cloth do you use a day?”*. They were also asked to report (*Yes/No*) if they shared cloth with anyone in their household. Girls provided yes/no responses to indicate whether or not they had underwear, and if they wore underwear with their menstrual absorbent.

**Cleaning and changing absorbents.** Girls provided a numbered response to the question: *“How many minutes does it take for you to change your sanitary protection?”.* They were asked if they were able to *“go for a full day without changing protection”* with response options “*no, never*”, “*yes, on some days*” and “*yes, always*”. Girls were asked to rate on a 3-point Likert scale if changing their absorbent at school was “*not a problem at all”, “a little bit of a problem”* or *“a big problem*”. Girls provided yes/no responses to items asking if they washed their absorbents, used soap, used clean water for washing, and if water was hot or cold. Girls also provided a yes/no response to the item *“Did you feel disgusted by washing the pad/cloth?”.* Absorbent drying time was reported as the number of hours the pad or cloth took to dry, and girls reported if they wore their absorbent damp “*never”, “sometimes”,* or “*often”.*

**Absorbent reliability.** Girls were asked: *“Do you feel you can rely on this method* [most frequently used absorbent] *to keep you from soiling when you have to…”* followed by the list of activities reported in results tables. They reported that leaking when using their absorbent was *“not at all a problem”, “a little bit of a problem”* or *“a big problem”*. Girls reported if they worried about odour: “*never”, “rarely”, “sometimes”, “often”* or “*all of the time”.* This was followed by a question asking girls to report on the situations in which they worried about odour. Girls provided yes/no responses to if they had experienced a list of events during their *last menstrual period*, including odour, and outside garment soiling.

**Restrictions due to menstruation.** Girls were asked if their menstruation *ever causes* them to miss or avoid certain activities. Interviewers read a list of activities such as such as physical exercise or being around males and girls reported if their menstruation caused them to miss or avoid this activity. Similarly, girls provided a yes/no response to the item: *“are there any activities or settings that you avoid while on your menstrual period?”*. For those who indicated that there were, girls were asked to list them. Girls were also asked: “do *the boys you know tease girls about their MP (menstrual period)”,* and provided yes/no responses.

**Reusable pad ratings.** For girls who reported that they had received reusable pads, items captured how much they liked them (from “*dislike very much*” to “*like it very much*” on a 4-points scale). Girls were asked how likely they would be to purchase such pads if they had the resources to do so (from *“not at all likely”* to *“extremely likely”* on a 5-point scale, with mid-point of *“moderately likely”*).

### Analyses

Analyses were conducted with Stata 14.0 [22]. Descriptive statistics captured participant characteristics and schoolgirls’ satisfaction with reusable pads. Description of girls’ experiences and reliability ratings are presented for each type of absorbent (reusable pads, new or old cloth, disposable sanitary pads, or other which included toilet paper, mattress, and underwear alone). However, there was insufficient power to compare differences in experience and reliability across the 5 absorbent types. It was not deemed appropriate to group commercial sanitary pads, as a purpose-built menstrual absorbent, with other improvised methods such as cloth. Thus, the existing improvised absorbents (new or old cloth, or other absorbents including toilet paper, mattress, and underwear alone) were compared with girls using the reusable pads received as part of the *Menstruating and the Cycle of Poverty* trial. Additional sensitivity analyses (available from corresponding author on request) included girls using disposable sanitary pads in the comparison group as a treatment-as-usual comparison and found no difference in the pattern of observed effects. Experiences of girls using disposable sanitary pads are retained in tables but there was insufficient power to test statistically significant differences for this group. Girls using reusable pads were compared to those using improvised methods using binary logistic regressions for dichotomous outcomes, and *t*-tests for continuous outcomes. Reference categories for logistic regressions were selected to favour positive odds ratios for more intuitive interpretation for readers.

## Results

### Participant characteristics

Girls’ self-reported age ranged from 10 to 19 years (*Mean* = 14.20,*SD =* 1.12), with the majority (86.9%) aged 13 to 15. The average age at menarche was 12.82 years (*SD =* 1.28). Most girls were in upper primary school (Primary class (P) 5: 21.5, P6: 44.9, P7: 24.9%) and attended the same school last year (94.6%). When asked how long it took them to get to school 37.7% reported an 11–30-min walk, 37.7% a 31–60-min walk, and smaller proportions reported a walk less than 10 min (19.8%) or longer than 1 h (4.8%).

### Menstrual absorbents

201 girls answered questions about the type of absorbent they used most frequently. Of these, 72 girls (35.8%) used reusable pads, 30 (14.9%) new cloth, 62 (30.9%) old cloth, 18 (9.0%) disposable sanitary pads, and 19 (9.5%) ‘other’ methods including toilet paper, underwear alone, mattress, or sponge. 100% of girls in the sample stated that they had underwear and 97.6% reported that they wore underwear with sanitary protection. Table 1 describes girls’ access to the different menstrual absorbents. For girls who did not receive reusable pads, new cloth was more often provided by mothers, and old cloth more often obtained by girls themselves. Girls using new cloth reported having more cloth, and using more pieces per day. Few girls reported sharing cloth.

**Table 1:** Schoolgirls’ self-reported access to absorbents

	Reusable pad *N* = 72	New Cloth *N* = 30	Old Cloth *N* = 62	Disposable Sanitary Pad *N* = 18	Other *N* = 19
	%(*N*)	%(*N*)	%(*N*)	%(*N*)	%(*N*)
Who provides you with your absorbent?					
Obtain myself	–	13.3 (4)	59.7 (37)	5.6 (1)	31.6 (6)
Mother	–	76.7 (23)	32.3 (20)	72.2 (13)	26.3 (5)
Other household member	–	6.7 (2)	8.1 (5)	5.6 (1)	10.5 (2)
AFRIpads (intervention)	100 (72)	–	–	–	–
Other (hospital doctor)	–	3.3 (1)		16.7 (3)	5.3 (1)
*Don’t know/no answer*					*26.3 (5)*
Anyone else in your house use this absorbent?					
Yes	–	53.3 (16)	45.2 (28)	72.2 (13)	36.8 (7)
No	–	40.0 (12)	50.0 (31)	27.8 (5)	36.8 (7)
*Missing or Not Applicable*	*100 (72)*	*6.7 (2)*	*4.8 (3)*	*0 (0)*	*26.3 (5)*
How many pieces of cloth do you have?					
1–2		7.7 (2)	25.9 (15)		
3–4		50.0 (13)	37.9 (22)		
5+		42.3 (11)	36.2 (21)		
How many pieces of cloth do you use a day?					
1–2		38.5 (10)	53.5 (31)		
3+ (max reported 6)		61.5 (16)	46.6 (27)		
Do you share cloth with anyone else in your household?					
Yes		0 (0)	3.4 (2)		
No		100 (26)	96.6 (57)		

### Experience of cleaning and changing menstrual absorbents

Table 2 displays girls’ changing and cleaning practices. The left side of the table compares reusable pads to existing improvised methods combined, while the right section of the table presents the proportion of behaviours broken down across absorbent types. Girls using reusable pads reported that it took them less time to change, and were more likely to report that it was “*not a problem at all”* to change at school. They were less likely to report that they could go a whole day without changing their absorbent.

**Table 2:** Experience of changing and cleaning absorbents compared between those using reusable pads and existing improvised methods

	Reusable pad *N* = 72	Existing improvised methods *N* = 111	OR (95%CI) (Existing method OR = 1.00)	Existing improvised methods (disaggregated)			Sanitary pad *N* = 18
				New Cloth *N* = 30	Old Cloth *N* = 62	Other *N* = 19	
	%(*N*)	%(*N*)		%(*N*)	%(*N*)	%(*N*)	%(*N*)
How many minutes does it take for you to change your sanitary protection? (*n* = 178)							
Minutes *M (SD)*	6.36 (11.75)	10.85 (13.96)	d = 0.35 (0.04–0.64)	9.17 (13.94)	11.46 (14.54)	11.61 (12.43)	10.44 (12.27)
0–4 min	69.6 (48)	49.5 (54)	2.24 (1.19–4.23)	53.3 (16)	50.8 (31)	44.4 (8)	44.4 (8)
5+ minutes	30.4 (21)	50.5 (55)	1.00	46.7 (14)	49.2 (30)	55.6 (10)	55.6 (10)
Are you able to go to school for a full day without changing your protection? (*n* = 180)							
No, never	70.8 (51)	50.9 (55)	1.00	53.3 (16)	49.2 (30)	52.9 (9)	61.1 (11)
Yes, on some days	13.9 (10)	23.2 (25)	0.43 (0.23–0.80)	16.7 (5)	31.2 (19)	5.9 (1)	11.1 (2)
Yes, always	15.3 (11)	25.9 (28)		30.0 (9)	19.7 (12)	41.2 (7)	27.8 (5)
How much of a problem is it to change your sanitary protection at school? (*n* = 140)^a^							
Not a problem at all	41.0 (25)	7.6 (6)	8.44 (3.18–22.43)	14.3 (3)	4.2 (2)	10.0 (1)	38.5 (5)
A little bit of a problem	29.5 (18)	32.9 (26)	1.00	38.1 (8)	31.3 (15)	30.0 (3)	46.2 (6)
A big problem	29.5 (18)	59.5 (47)		47.6 (10)	64.6 (31)	60.0 (6)	15.4 (2)
Did you feel disgusted by washing the absorbent? (*n* = 154)^b^							
Yes	22.5 (16)	48.2 (40)	1.00	65.4 (17)	40.4 (23)		
No	77.5 (55)	51.8 (43)	3.20 (1.58–6.46)	34.6 (9)	59.7 (34)		
How long did it take the absorbent to dry completely? (*n* = 151)^b^							
2 h or less	26.1 (18)	48.8 (40)	1.00	50.0 (13)	48.2 (27)		
3–4 h	30.4 (21)	29.3 (24)		23.1 (6)	32.1 (18)		
5–6 h	26.6 (17)	11.0 (9)	2.74 (1.35–5.55)	11.5 (3)	10.8 (6)		
7 h +	18.8 (13)	11.0 (9)		15.4 (4)	8.9 (5)		
How often did you wear the absorbent damp? (*n* = 154)^b^							
Never	85.9 (61)	69.9 (58)	2.63 (1.16–5.95)	61.5 (16)	73.7 (42)		
Sometimes	6.9 (5)	6.0 (5)	1.00	11.5 (3)	3.5 (2)		
Usually	6.9 (5)	24.1 (20)		26.9 (7)	22.8 (13)		

^a^Of girls who reported they could not go a whole day without changing their absorbent at school

^b^Of girls who reported washing and reusing their absorbent, and not asked of girls using disposable sanitary pads or ‘other’ absorbent

Of girls using reusable absorbents (AFRIpads, cloth), almost all (98.0%) reported washing them. Of those, 98.0% reported using soap every time. Almost all (98.7%) reported using clean water, and most reported that it was cold (89.0%). Girls using the reusable pads were more likely to report that they were not disgusted by washing their absorbent, in contrast to those using cloth. Although those using the pads reported longer drying times, they were more likely to report never wearing them damp.

### Absorbent reliability

Girls’ ratings of perceived reliability and their experience of using their absorbent are displayed in Table 3. Again, the left side of the table displays comparison between those using the reusable pads and a pooled total of those using existing improvised methods, which are broken down by absorbent type in the right section of the table. The ratings for the few girls using commercial disposable sanitary pads are displayed in the far right column for comparison.

**Table 3:** Schoolgirls’ ratings of absorbent reliability compared between those using reusable pads and those using existing improvised methods

	Reusable pad *N* = 72	Existing improvised methods *N* = 111	OR (95%CI) (Existing method OR = 1.00)	Existing improvised methods (disaggregated)			Sanitary pad *N* = 18
				New Cloth *N* = 30	Old Cloth *N* = 62	Other *N* = 19	
	%(*N*)	%(*N*)		%(*N*)	%(*N*)	%(*N*)	%(*N*)
Do you feel you can rely on this absorbent to keep you from soiling when you have to…							
Walk a long distance	75.7 (53)	37.7 (40)	5.14 (2.62–10.08)	43.3 (13)	37.1 (23)	28.6 (4)	88.9 (16)
Be gone from home for a long time	75.7 (53)	40.6 (43)	4.31 (2.23–8.35)	40.0 (12)	40.3 (25)	42.9 (6)	77.8 (14)
Move quickly or strenuously	73.6 (53)	49.1 (52)	2.90 (1.52–5.54)	46.7 (14)	46.8 (29)	64.3 (9)	72.2 (13)
Sit for a long time	68.1 (49)	35.9 (38)	3.81 (2.02–7.19)	23.3 (7)	38.7 (2)	50.0 (7)	77.8 (14)
Be around males	59.2 (42)	28.6 (30)	3.62 (1.92–6.83)	27.6 (8)	25.8 (16)	42.9 (6)	66.7 (12)
Go to the farm	88.6 (62)	60.0 (63)	5.17 (2.25–11.89)	55.2 (16)	61.3 (38)	64.3 (9)	100 (16)
Go to school	90.1 (64)	53.8 (57)	7.86 (3.30–18.74)	60.0 (18)	51.6 (32)	50.0 (7)	100 (17)
Go to the market	83.3 (60)	43.4 (46)	6.52 (3.15–13.52)	46.7 (14)	40.3 (25)	50.0 (7)	88.2 (15)
How much of a problem is leaking when using this absorbent? (*n* = 174)							
Not a problem at all	55.6 (40)	14.71 (15)	7.25 (3.53–14.87)	4.6 (1)	14.8 (9)	38.5 (5)	72.2 (13)
A little bit of a problem	34.7 (25)	35.3 (36)	1.00	64.3 (18)	27.9 (17)	7.7 (1)	22.2 (4)
A big problem	9.7 (7)	50.0 (51)		32.1 (9)	57.4 (35)	53.9 (7)	5.6 (1)
Do you often worry about odour? (*n* = 181)							
Never	42.3 (30)	40.0 (44)	1.10 (0.60–2.01)	40.0 (12)	41.0 (25)	36.8 (7)	38.9 (7)
Rarely/Sometimes	19.7 (14)	29.1 (32)	1.00	23.3 (7)	32.8 (20)	26.3 (5)	44.4 (8)
Often/All the time	38.0 (27)	30.9 (34)		36.7 (11)	26.2 (16)	36.8 (7)	16.7 (3)
During your last menstrual period did you experience… (*n* = 140)^a^							
Fear of panty soiling	60.0 (33)	54.1 (46)	1.27 (0.64–2.53)	54.6 (12)	53.2 (25)	56.3 (9)	61.5 (8)
Odour	29.1 (16)	32.9 (28)	0.84 (0.40–1.74)	31.8 (7)	38.3 (18)	18.8 (3)	23.1 (3)
Outside garment soiling	43.6 (24)	43.5 (37)	1.00 (0.51–1.99)	45.5 (10)	44.7 (21)	37.5 (6)	61.5 (8)
Fear that sanitary protection would fall out of underwear	47.3 (26)	49.4 (42)	0.92 (0.47–1.81)	63.6 (14)	46.8 (22)	37.5 (6)	23.1 (3)

^a^An error in the survey application meant in 38 cases this item failed to load, a further 10 girls did not respond

Girls using reusable pads reported significantly higher perceptions of reliability across all listed activities than those using existing methods. They were also much more likely to report that leaking was “*not a problem at all”*. Despite large differences in the self-reported belief that they could rely on the reusable pads to keep them from soiling across situations, there were no differences in reports of experiencing actual panty or outer garment soiling, odour, or fear that sanitary protection would fall out of underwear. There were also no differences in reported concerns about odour. Follow-up items (not included in the table) found that for those who did worry about odour (*n* = 117), they worried about odour at school (87.2%), around males (39.3%) and in other environments (e.g., church, market) (53.9%).

### Freedom to participate in daily activities

A total of 116 girls (63.4%, excluding those using sanitary pads) reported that there were activities or settings they avoided when menstruating. Table 4 presents the activities girls reported missing due to menstruation as well as activities or settings they avoid, according to menstrual absorbent. There were no significant differences in the daily activities girls reported their menstruation caused them to miss. Similarly, there was no difference in girls reporting that there were activities or settings they avoided while menstruating. Of those who did report avoiding activities or settings, girls using existing methods were more likely to report avoiding physical sports or exercise, working in the field or garden, fetching water, or cooking. There were no differences in reported avoidance of being around males, playing with other children, or other going to school. Despite a large (almost 10%) difference between the proportion of girls reporting boys teased girls about their menstruation, favouring existing methods, this difference was not significant (although trended towards significance, *p* = .208).

**Table 4:** Comparison of participation in daily activities between reusable pads and existing improvised methods

	Reusable pad *N* = 72	Existing improvised methods *N* = 111	OR (95%CI) (Reusable pad OR = 1.00)
	%(*N*)	%(*N*)	
Does your menstruation ever cause you to: (*n* = 169)			
Miss school	17.2 (11)	21.9 (23)	1.35 (0.61–3.00)
Not do your homework	6.3 (4)	8.6 (9)	1.41 (0.41–4.77)
Miss work in the field	17.2 (11)	21.0 (22)	1.28 (0.57–2.85)
Be unable to play with other children	37.5 (24)	41.9 (44)	1.20 (0.64–2.27)
Avoid physical sports/exercise	37.5 (24)	43.8 (46)	1.30 (0.69–2.45)
Avoid being around males	60.9 (39)	49.5 (52)	0.63 (0.33–1.18)
Avoid chores	7.8 (5)	5.7 (6)	0.72 (0.21–2.45)
Avoid sex	39.1 (25)	41.9 (44)	1.13 (0.60–2.12)
Are there activities or settings that you avoid while menstruating? (*n* = 183)			
Yes	67.6 (48)	61.8 (68)	1.00
No	32.4 (23)	38.2 (42)	1.29 (0.69–2.41)
*No answer*	*1*	*1*	
Activity or setting (*n* = 116)^a^			
Serving food and beverages to guests	6.3 (3)	11.8 (8)	2.00 (0.50–7.97)
Being around males	56.3 (27)	45.6 (31)	0.65 (0.31–1.37)
Fetching water	18.8 (9)	41.2 (28)	3.03 (1.27–7.25)
Cooking	8.3 (4)	26.5 (18)	3.96 (1.25–12.59)
Being in a sacred space	0 (0)	7.4 (5)	–
Physical sports/exercise	10.4 (5)	36.8 (25)	5.00 (1.75–14.28)
Playing with other children	35.4 (17)	38.2 (26)	1.13 (0.52–2.43)
Working in the field/garden	2.1 (1)	29.4 (20)	19.58 (2.53–151.85)
Going to school	6.3 (3)	8.8 (6)	1.45 (0.34–6.12)
Doing homework	0 (0)	4.4 (3)	–
Do the boys you know tease girls about their menstruation? (*n* = 183)			
Yes	52.8 (38)	43.2 (48)	1.00
No	47.2 (34)	56.8 (63)	1.47 (0.81–2.66)

^a^Of girls who reported there were activities or settings they avoided while menstruating

### Reusable pad use and appraisal

While 72 girls in the sample reported most frequently using the reusable pads as their absorbent, a further four received but never used them, five received the pads and used them a few times before discontinuing use, and 14 received the reusable pads and continued using them (but did not report them as their main absorbent). Of the 23 girls who reported using reusable pads but did not report these as their primary menstrual absorbent, six were using new cloth, eight were using old cloth, two were using sanitary pads and two were using other methods.

Of girls who never used the reusable pads, one reported this was because they were stolen, two because they trusted regular pads more, and one because she did not know how to dispose of them. Of those who discontinued pad use, two reported that they preferred regular disposable pads, 1 that she had lost them, one that she felt the reusable pads burned, and 1 because they “*did not look like they would work well”*. No items captured reasons girls most frequently used alternate absorbents for the 14 who had not reported discontinued reusable pad use.

Table 5 displays girls’ appraisal of the reusable pads provided. The table includes girls who reported reusable pads as their usual absorbent, as well as those who had received and used them at least once.

**Table 5:** Schoolgirls’ appraisal of the reusable pads (*n* = 91)

	Percent	Number
How much did you like the AFRIpad?		
Dislike it very much	2.2	2
Dislike it a little	1.1	1
Like it a little	4.4	4
Like it very much	92.3	84
AFRIpads cost 15,000 shillings (UGX)^a^ and last for a year. Would you be able to afford them?		
No	52.3	46
Maybe	2.3	2
Yes	45.5	40
If you had 15,000 shillings, how likely would you be to buy the AFRIpads?		
Not at all likely	3.4	3
Slightly likely	14.8	13
Moderately likely	1.1	1
Very likely	37.5	33
Extremely likely	43.2	38

^a^for reference, in the local area: school fees for one term 10,000 UGX, exercise book 200 UGX, soap (small piece) 500 UGX, soap (regular bar) 2000 UGX, laundry detergent (small bag) 2500 UGX, petticoat 1500 UGX, maize flour 1 kg 1500 UGX, rice 1 kg 3000 UGX

## Discussion

The present study was the most comprehensive quantitative assessment to date comparing girls’ experiences of different menstrual absorbents in a low income context. Specifically, comparing reusable sanitary pads to existing improvised methods, predominantly cloth, in rural Uganda. The reusable pads tested here, AFRIpads, received favourable ratings. They were reported to be quicker to change, less of a problem to change at school, and less disgusting to wash than other absorbents. Although these pads took longer to dry, girls were less likely to report wearing them damp. This may be a true difference as a result of the accompanying instruction not to wear them damp, or may represent social desirability bias in reporting, as girls knew they were not supposed to wear the pads damp.

Girls reported that they felt the reusable pads were more reliable to keep them from soiling in a variety of settings. Perceptions of reliability across ranged from 68 to 90% for those using reusable pads compared to 29–54% for those using existing improvised methods. Interestingly, among the highest ratings of 90 and 54% reliability were for protection from soiling when going to school. This is highly suggestive of girls providing biased, desired responses. Girls reported the lowest reliability of absorbents when sitting for a long time (68 and 36% for pads and existing improvised methods, respectively), one of the main school activities. Similarly, girls rated reliability when they needed to be gone from home for a long time as 76 and 41% for reusable pads and improvised methods, another aspect of the school day which did not corroborate girls’ ratings of absorbent reliability when at school. As AFRIpads were the only product provided in the intervention, this study was unable to test if girls would have rated other provided products more highly (e.g., other types of reusable pad, reusable menstrual underwear, tampons). Only a very small group used disposable sanitary pads, which may have been favoured and showed high ratings. No girls reported using inserted methods. Girls reported liking the reusable pads, although half reported that they would not be able to afford them. Girls stated they would be likely to spend money on these products if they had the funds available but they were not asked if they would favour other types of products; an important consideration for future research.

The above ratings indicate positive perceptions of reliability of the reusable pads, and may indicate better performance. However, they also highlight issues with current outcome assessment in menstrual management research and discrepancies between girls’ perceptions of absorbents and their lived experience [13, 21]. While girls using reusable pads had 8.44 times higher odds of reporting leaking was not a problem and reported higher perceptions of reliability, there were no differences in reported outer garment soiling during the last menses (44% for both those using reusable pads and those using improvised methods). These high proportions suggest that the provision of better absorbents may not be sufficient to protect against embarrassment. More research is needed on the frequency of soiling and contributing factors (e.g., wearing pads for too long, underwear quality, inadequate absorbents, being unprepared for menstruation and soiling when menstruation commences). It is also difficult to determine from these results if the reusable pads were more reliable absorbents, or only perceived to be so. Future work which collects objective measures of absorbency and fluid retention may answer these questions with regard to product development. Improved outcome measures may capture the time and causes of soiling which may also distinguish between absorbent failure and behavioural predictors of soiling. Similar rates of odour and concern about odour were reported for reusable pads and other absorbents. This could reflect other aspects of menstrual hygiene management such as washing and drying, which were not improved by pad provision [21]. Differences between perceptions of reliability and actual soiling and odour also highlight the value of quantiative and mixed-methods approaches to assessing absorbents as such discrepancies may not have been identified in qualitative studies.

Girls reported missing activities due to menstruation. Differences in responses to items *‘does your menstruation ever cause you to miss/avoid…’* where the list of items was read aloud, and *‘are there activities you avoid while menstruating…’* with volunteered responses, further demonstrates the need for more careful and validated outcome assessment in this field. Validity and reliability studies are needed, with lessons from fields with more research such as sexual and reproductive health which also ask for sensitive information about personal experience and practices, with known social desirability. Across a range of domains there is a need for measure development. Further, mixed-methods research may assist in attaining the nuanced measurement of proposed outcomes. Despite issues of outcome assessment, comparison between reusable pads and existing methods revealed fewer differences than expected in daily activities. When girls were asked if their menstruation ever caused them to miss certain activities there were no differences in reports. When asked to volunteer settings avoided while menstruating there were large differences between pads and existing methods on selected items. This may indicate differences in volunteered responses, or being asked if they avoided activities, in contrast to asking if menstruation caused them to miss activities. Girls using existing methods had 5.00 times higher odds of avoiding physical sports and exercise, and were more likely to report avoiding fetching water, cooking or working in the field or garden.

Together study findings reveal the importance of considering the full story of menstrual management when assessing interventions. Primary trial outcomes reporting the same improvements in school attendance following reusable pad provision as puberty education, [14] do not reveal improvements to girls’ confidence in absorbents, disgust in washing, difficulties changing absorbents, or participation in other activities. Findings also suggest that the provision of pads alone may not be sufficient to reduce garment soiling and that more detailed work is needed.

Although not statistically significant, there was an almost 10% higher report of teasing amongst those using the trial-provided reusable pads. No trials to date have assessed potential harms of menstrual management interventions in low income contexts, [5] this finding cautions that the provision of sanitary products in schools may leave girls vulnerable to teasing about menstruation if interventions draw attention to, or ‘outs’, menstruating girls.

### Strengths and limitations

This study reported on a sample of girls in rural Uganda. Although the work is cross-sectional, the reusable pads were provided as part of a quasi-randomised trial to girls in 4 of the 8 study schools. Socio-demographic differences are likely to exist between those using the range of other existing methods, however as the reusable pads were distributed free as part of the trial these should not vary according to socio-demographics. It should also be noted that girls who were not included in the trial, but moved to intervention schools by March 2014 may also have received pads or have been surveyed and so were included here. When pads were delivered, they were provided to all menstruating girls as not to discriminate or create known inequality between students. With regard to generalisability of the findings, results of the main trial found those attending school at follow-up and therefore completing follow-up surveys had higher attendance than those who had dropped out by this point [14]. Thus the present sample is likely to have higher attendance and more favourable circumstances than those who had dropped out of school already.

The use of local research assistants to administer surveys was advantageous in use of the local language and access to the population. Given the low literacy rate in the area, verbal administration of the survey was likely to be more reliable than written surveys, which would have also required costly translation and back-translation efforts. However, this method was vulnerable to social-desirability in girls’ reports, as noted above. Social-desirability in reporting may have been exacerbated by asking questions in the school setting. Although girls were taken aside to ask questions there were many others around which may have primed social norms and led to biases towards expected responses. The local NGO was well known and respected in the area, with recent high-profile campaigns for girls’ education (https://www.plan-international.org/what-we-do/because-i-am-girl). Many interviewing research assistants were the same as those who had provided the pads, which may have also biased girls towards favourable reports. These biases are likely to exist in most studies in this area and must be considered in interpreting results.

The sample size of this study was insufficient to assess outcomes across all absorbents. Frequencies suggest that disposable pads may have been more favourable than reusable pads in some areas, but there was insufficient power to compare these. Future studies are needed, with larger sample sizes, and should seek to compare a variety of absorbents. Further, future cross-sectional comparisons would be improved by adjusting for potential socio-demographic confounds.

## Conclusions

Results of this study suggest reusable pads are perceived to be more reliable and convenient to manage than girls’ existing improvised menstrual absorbents (predominantly cloth) in rural Uganda. Whilst the primary outcomes of the trial found no difference between reusable pads and puberty education in improving school attendance, this follow-up work reveals other important benefits of pad provision. Findings suggest that to fully appraise programs providing sanitary products to girls, a wide range of outcomes must be considered. Girls’ comfort and dignity must be kept in the forefront of evaluation and consideration by policy makers when interpreting the results of existing studies. Potential harms from outing girls’ menstrual status, or reductions to their choice of menstrual absorbents by restricting them to only one advised product should also be considered. Ideally schoolgirls may be provided with a choice of products, and willingness and ability to pay studies may provide further details on preferences and practices.

## Additional files

Additional file 1: STROBE 2007 (v4) Statement—Checklist of items that should be included in reports of *cross-sectional studies. (DOCX 21 kb)*

Additional file 2: Intervention Survey Oct 2014. (PDF 236 kb)
