# Supplementary material for: Schoolgirls’ experience and appraisal of menstrual absorbents in rural Uganda: a cross-sectional evaluation of reusable sanitary pads
Source: Reprod Health. 2016 Dec 7;13:143. doi: 10.1186/s12978-016-0260-7 (PMC5142137; doi:10.1186/s12978-016-0260-7)
Supplement: Additional file 2: — Intervention Survey Oct 2014. (PDF 236 kb) [file 12978_2016_260_MOESM2_ESM.pdf]

Intervention Survey Oct 2014

Introduction

You may remember that we came here last year and interviewed you about your MP for a study meant to find out how best to help girls manage their MP while they are in school. If you choose to participate in the study again today, we will ask you similar questions about your menstruation. The questions will take about 30 minutes. We will never reveal your identity to anyone. In fact, all your answers will be kept secret and we are not going to tell anyone. You may choose to participate or not and may quit at any time. Would you like to continue? Your name will not be associated with the picture and will only be used in academic presentations, lectures and/or publications associated with this project.

Interview consent:*
[RA: TICK THE APPROPRIATE 'YES' BOX IF CONSENT HAS BEEN EXPLAINED AND RESPONDENT HAS AGREED. IF CONSENT HAS NOT BEEN OBTAINED, DO NOT CONTINUE AND CONTACT YOUR SUPERVISOR FOR INSTRUCTIONS]
( ) Yes, consent obtained
( ) No (consent NOT obtained)

Study ID:* ____

General Demographics Information

Logic: Show/hide trigger exists.

Page 2 - [RA: PLEASE SELECT AN INTERVIEW DISPOSITION.]*
( ) Girl consented to interview. - *This will enable more questions below.*
( ) Consent was <u>not</u> obtained.

Logic: Hidden unless: Question "Page 2 - [RA: PLEASE SELECT AN INTERVIEW DISPOSITION.]" is one of the following answers ("Girl consented to interview. - *This will enable more questions below.*")

Okay, here is the first question. How old are you?*

[ENTER NUMBER] [IF GIRL DOESN'T KNOW ENTER DK] [IF NO ANSWER ENTER 999]

____

Logic: Hidden unless: Question "Page 2 - [RA: PLEASE SELECT AN INTERVIEW DISPOSITION.]" is one of the following answers ("Girl consented to interview. - *This will enable more questions below.*")

What class in school are you?*
( ) P3
( ) P4
( ) P5
( ) P6
( ) P7
( ) NONE OF THESE/NO ANSWER

Logic: Show/hide trigger exists. Hidden unless: Question "Page 2 - [RA: PLEASE SELECT AN INTERVIEW DISPOSITION.]" is one of the following answers ("Girl consented to interview. - *This will enable more questions below.*")

Did you go to the same school last year?*
( ) Yes
( ) No (trigger)
( ) No answer

Logic: Hidden unless: Question "Did you go to the same school last year?" #3 is one of the following answers ("No (trigger)")

Which school did you go to?*

____

Logic: Hidden unless: Question "Page 2 - [RA: PLEASE SELECT AN INTERVIEW DISPOSITION.]" is one of the following answers ("Girl consented to interview. - *This will enable more questions below.*")

Are you repeating this class?*
( ) Yes
( ) No
( ) No answer

Logic: Hidden unless: Question "Page 2 - [RA: PLEASE SELECT AN INTERVIEW DISPOSITION.]" is one of the following answers ("Girl consented to interview. - *This will enable more questions below.*")

Have you ever repeated any other class?*
( ) Yes
( ) No
( ) No answer

Empowerment

Logic: Show/hide trigger exists.

Page 3 - [RA: Please select an interview disposition.]*
( ) Girl consented to interview. - *This will enable more questions below*
( ) Consent was <u>not</u> obtained.

Logic: Hidden unless: Question "Page 3 - [RA: Please select an interview disposition.]" is one of the following answers ("Girl consented to interview. - *This will enable more questions below*")

[READ] Now, I am going to ask you for your own opinions on some things. For each question, please tell me the answer that comes closest to your own view.

Logic: Hidden unless: Question "Page 3 - [RA: Please select an interview disposition.]" is one of the following answers ("Girl consented to interview. - *This will enable more questions below*")

What do you think is the biggest factor in determining your future? Do you think it is...[READ]*
( ) Your own hard work

( ) Your family
( ) Your future husband
( ) Your community
( ) Your education
( ) Your government
( ) Or something else? (specify): ____*
( ) [DO NOT READ] DON'T KNOW/NO ANSWER

Logic: Hidden unless: Question "Page 3 - [RA: Please select an interview disposition.]" is one of the following answers ("Girl consented to interview. - *This will enable more questions below*")

Do you think education will increase your opportunities in life a little? A lot? Or not at all?*
( ) Increase my opportunities a little
( ) Increase my opportunities a lot
( ) Not increase my opportunities at all
( ) DON'T KNOW/NO ANSWER

Logic: Show/hide trigger exists. Hidden unless: Question "Page 3 - [RA: Please select an interview disposition.]" is one of the following answers ("Girl consented to interview. - *This will enable more questions below*")

Do you think you will be going to secondary school?*
( ) Will go
( ) Probably won't go (trigger)
( ) Other (specify): ____*
( ) DON'T KNOW/NO ANSWER

Logic: Hidden unless: Question "Do you think you will be going to secondary school?" #9 is one of the following answers ("Probably won't go (trigger)")

Why do you think you won't be going to secondary school?
[CHECK ALL THAT APPLY]*
[ ] I was not a good enough student
[ ] Parents refuse to pay for it
[ ] I have to work hard to sustain myself for food and clothes
[ ] Marriage
[ ] I might get pregnant
[ ] I won't perform well on the exam
[ ] No one wants me to go or encourages me
[ ] I just don't want to go
[ ] Other (specify): ____*
[ ] DON'T KNOW/NO ANSWER

Logic: Show/hide trigger exists. Hidden unless: Question "Page 3 - [RA: Please select an interview disposition.]" is one of the following answers ("Girl consented to interview. - *This will enable more questions below*")

Do you think you will be able to finish primary school?*
( ) Think I will stay through the end of P7
( ) Think I will likely leave (trigger)
( ) DON'T KNOW/NO ANSWER

Logic: Hidden unless: Question "Do you think you will be able to finish primary school?" #11 is one of the following answers ("Think I will likely leave (trigger)")

Why do you think you will likely leave?
[CHECK ALL THAT APPLY]*
[ ] I was not a good enough student
[ ] Parents refuse to pay for it
[ ] I have to work hard to sustain myself for food and clothes
[ ] Marriage
[ ] I might get pregnant
[ ] I won't perform well on the exam
[ ] No one wants me to go or encourages me
[ ] I just don't want to go
[ ] Other (specify): ____*
[ ] DON'T KNOW/NO ANSWER

Logic: Hidden unless: Question "Page 3 - [RA: Please select an interview disposition.]" is one of the following answers ("Girl consented to interview. - *This will enable more questions below*")

Do you like going to school?*
( ) Like going to school
( ) Would rather not go to school
( ) DON'T KNOW/NO ANSWER

Logic: Hidden unless: Question "Page 3 - [RA: Please select an interview disposition.]" is one of the following answers ("Girl consented to interview. - *This will enable more questions below*")

Going to school is more important for boys? Or girls? Or is it the same for both?*
( ) Boys
( ) Girls
( ) The same for both
( ) DON'T KNOW/NO ANSWER

Subjective Well-being

Logic: Show/hide trigger exists.

Page 4 - [RA: Please select an interview disposition.]*
( ) Girl consented to interview. - *This will enable more questions below.*
( ) Consent was <u>not</u> obtained.

Logic: Hidden unless: Question "Page 4 - [RA: Please select an interview disposition.]" is one of the following answers ("Girl consented to interview. - *This will enable more questions below.*")

[READ] Now, I want you to think about your life over the past six months. I am going to read some sentences. For each one, please tell me if it is not true, sometimes true, or true about you.

Logic: Hidden unless: Question "Page 4 - [RA: Please select an interview disposition.]" is one of the following answers ("Girl consented to interview. - *This will enable more questions below.*")

Here is the first sentence: I try to be nice to other people. I care about their feelings. Is that...[READ]*
( ) Not true
( ) Sometimes true
( ) True
( ) [DO NOT READ] DON'T KNOW/NO ANSWER

Logic: Hidden unless: Question "Page 4 - [RA: Please select an interview disposition.]" is one of the following answers ("Girl consented to interview. - *This will enable more questions below.*")

I am restless. I cannot stay still for long. Is that...[READ]*

( ) Not true
( ) Sometimes true
( ) True
( ) [DO NOT READ] DON'T KNOW/NO ANSWER

Logic: Hidden unless: Question "Page 4 - [RA: Please select an interview disposition.]" is one of the following answers ("Girl consented to interview. - *This will enable more questions below.*")

I get a lot of headaches, stomach aches, or sickness. Is that...[READ]*

( ) Not true
( ) Sometimes true
( ) True
( ) [DO NOT READ] DON'T KNOW/NO ANSWER

Logic: Hidden unless: Question "Page 4 - [RA: Please select an interview disposition.]" is one of the following answers ("Girl consented to interview. - *This will enable more questions below.*")

I usually share with others, for example, games, food. [READ IF NECESSARY]*

( ) Not true
( ) Sometimes true
( ) True
( ) [DO NOT READ] DON'T KNOW/NO ANSWER

Logic: Hidden unless: Question "Page 4 - [RA: Please select an interview disposition.]" is one of the following answers ("Girl consented to interview. - *This will enable more questions below.*")

I get very angry and often lose my temper. [READ IF NECESSARY]*

( ) Not true
( ) Sometimes true
( ) True
( ) [DO NOT READ] DON'T KNOW/NO ANSWER

Logic: Hidden unless: Question "Page 4 - [RA: Please select an interview disposition.]" is one of the following answers ("Girl consented to interview. - *This will enable more questions below.*")

I would rather be alone than with people of my age. [READ IF NECESSARY]*

( ) Not true
( ) Sometimes true
( ) True
( ) [DO NOT READ] DON'T KNOW/NO ANSWER

Logic: Hidden unless: Question "Page 4 - [RA: Please select an interview disposition.]" is one of the following answers ("Girl consented to interview. - *This will enable more questions below.*")

I usually do as I am told. [READ IF NECESSARY]*

( ) Not true
( ) Sometimes true
( ) True
( ) [DO NOT READ] DON'T KNOW/NO ANSWER

Logic: Hidden unless: Question "Page 4 - [RA: Please select an interview disposition.]" is one of the following answers ("Girl consented to interview. - *This will enable more questions below.*")

I am helpful if someone is hurt, upset or feeling ill. [READ IF NECESSARY]*
( ) Not true
( ) Sometimes true
( ) True
( ) [DO NOT READ] DON'T KNOW/NO ANSWER

Logic: Hidden unless: Question "Page 4 - [RA: Please select an interview disposition.]" is one of the following answers ("Girl consented to interview. - *This will enable more questions below.*")

I have one good friend or more. [READ IF NECESSARY]*
( ) Not true
( ) Sometimes true
( ) True
( ) [DO NOT READ] DON'T KNOW/NO ANSWER

Logic: Hidden unless: Question "Page 4 - [RA: Please select an interview disposition.]" is one of the following answers ("Girl consented to interview. - *This will enable more questions below.*")

I fight a lot. [READ IF NECESSARY]*
( ) Not true
( ) Sometimes true
( ) True
( ) [DO NOT READ] DON'T KNOW/NO ANSWER

Logic: Hidden unless: Question "Page 4 - [RA: Please select an interview disposition.]" is one of the following answers ("Girl consented to interview. - *This will enable more questions below.*")

I can make other people do what I want. [READ IF NECESSARY]*
( ) Not true
( ) Sometimes true
( ) True
( ) [DO NOT READ] DON'T KNOW/NO ANSWER

Logic: Hidden unless: Question "Page 4 - [RA: Please select an interview disposition.]" is one of the following answers ("Girl consented to interview. - *This will enable more questions below.*")

I am often unhappy, depressed, or tearful. [READ IF NECESSARY]*
( ) Not true
( ) Sometimes true
( ) True
( ) [DO NOT READ] DON'T KNOW/NO ANSWER

Logic: Hidden unless: Question "Page 4 - [RA: Please select an interview disposition.]" is one of the following answers ("Girl consented to interview. - *This will enable more questions below.*")

Other people my age generally like me. [READ IF NECESSARY]*
( ) Not true
( ) Sometimes true
( ) True
( ) [DO NOT READ] DON'T KNOW/NO ANSWER

Logic: Hidden unless: Question "Page 4 - [RA: Please select an interview disposition.]" is one of the following answers ("Girl consented to interview. - *This will enable more questions below.*")

I am easily distracted. I find it difficult to concentrate. [READ IF NECESSARY]*
( ) Not true
( ) Sometimes true
( ) True
( ) [DO NOT READ] DON'T KNOW/NO ANSWER

Logic: Hidden unless: Question "Page 4 - [RA: Please select an interview disposition.]" is one of the following answers ("Girl consented to interview. - *This will enable more questions below.*")

I am nervous in new situations. I can easily lose confidence. [READ IF NECESSARY]*
( ) Not true
( ) Sometimes true
( ) True
( ) [DO NOT READ] DON'T KNOW/NO ANSWER

Logic: Hidden unless: Question "Page 4 - [RA: Please select an interview disposition.]" is one of the following answers ("Girl consented to interview. - *This will enable more questions below.*")

I am kind to younger children. [READ IF NECESSARY]*
( ) Not true
( ) Sometimes true
( ) True
( ) [DO NOT READ] DON'T KNOW/NO ANSWER

Logic: Hidden unless: Question "Page 4 - [RA: Please select an interview disposition.]" is one of the following answers ("Girl consented to interview. - *This will enable more questions below.*")

I am often accused of lying or cheating. [READ IF NECESSARY]*
( ) Not true
( ) Sometimes true
( ) True
( ) [DO NOT READ] DON'T KNOW/NO ANSWER

Logic: Hidden unless: Question "Page 4 - [RA: Please select an interview disposition.]" is one of the following answers ("Girl consented to interview. - *This will enable more questions below.*")

Other children or young people pick on or bully me. [READ IF NECESSARY]*
( ) Not true
( ) Sometimes true
( ) True
( ) [DO NOT READ] DON'T KNOW/NO ANSWER

Logic: Hidden unless: Question "Page 4 - [RA: Please select an interview disposition.]" is one of the following answers ("Girl consented to interview. - *This will enable more questions below.*")

I often offer to help others (parents, teachers, children). [READ IF NECESSARY]*
( ) Not true
( ) Sometimes true
( ) True
( ) [DO NOT READ] DON'T KNOW/NO ANSWER

Logic: Hidden unless: Question "Page 4 - [RA: Please select an interview disposition.]" is one of the following answers ("Girl consented to interview. - *This will enable more questions below.*")

I think before I do things. [READ IF NECESSARY]*
( ) Not true
( ) Sometimes true
( ) True
( ) [DO NOT READ] DON'T KNOW/NO ANSWER

Logic: Hidden unless: Question "Page 4 - [RA: Please select an interview disposition.]" is one of the following answers ("Girl consented to interview. - *This will enable more questions below.*")

I take things that are not mine from home, school or elsewhere. [READ IF NECESSARY]*
( ) Not true
( ) Sometimes true
( ) True
( ) [DO NOT READ] DON'T KNOW/NO ANSWER

Logic: Hidden unless: Question "Page 4 - [RA: Please select an interview disposition.]" is one of the following answers ("Girl consented to interview. - *This will enable more questions below.*")

I get along better with adults than with people my own age. [READ IF NECESSARY]*
( ) Not true
( ) Sometimes true
( ) True
( ) [DO NOT READ] DON'T KNOW/NO ANSWER

Logic: Hidden unless: Question "Page 4 - [RA: Please select an interview disposition.]" is one of the following answers ("Girl consented to interview. - *This will enable more questions below.*")

I have many fears. I am easily scared. [READ IF NECESSARY]*
( ) Not true
( ) Sometimes true
( ) True
( ) [DO NOT READ] DON'T KNOW/NO ANSWER

Logic: Hidden unless: Question "Page 4 - [RA: Please select an interview disposition.]" is one of the following answers ("Girl consented to interview. - *This will enable more questions below.*")

I finish the work I'm doing. My attention is good. [READ IF NECESSARY]*
( ) Not true
( ) Sometimes true
( ) True
( ) [DO NOT READ] DON'T KNOW/NO ANSWER

Menstruation and Reproductive Health

Logic: Show/hide trigger exists.

Page 5 - [RA: Please select an interview disposition.]*
( ) Girl consented to interview. - *This will enable more questions below.*
( ) Consent was not obtained.

Logic: Hidden unless: Question "Page 5 - [RA: Please select an interview disposition.]" is one of the following answers ("Girl consented to interview. - *This will enable more questions below.*")

[READ] Now, I am going to ask you some questions about women's health and how you understand it. Please remember that we will not tell anyone else your answers.

Logic: Hidden unless: Question "Page 5 - [RA: Please select an interview disposition.]" is one of the following answers ("Girl consented to interview. - *This will enable more questions below.*")

Can you describe to me what MP is?*
[ENSURE GIRL GIVES <u>AT LEAST TWO ITEMS</u> ABOUT MP, BLOOD, PREGNANCY, PUBERTY ITEMS SUCH AS BREASTS]
( ) Yes, respondent understands
( ) No, respondent does not understand
( ) No answer

Logic: Show/hide trigger exists. Hidden unless: Question "Page 5 - [RA: Please select an interview disposition.]" is one of the following answers ("Girl consented to interview. - *This will enable more questions below.*")

Have you started your MP?*
( ) Yes - *This will enable more questions below.*
( ) No
( ) DON'T KNOW/NO ANSWER

Logic: Hidden unless: Question "Have you started your MP?" #40 is one of the following answers ("Yes - *This will enable more questions below.*")

When did you first start your MP?*
[ENTER YEAR] [IF EXACT MONTH NOT KNOWN, PROBE FOR ESTIMATED MONTH.] [IF GIRL DOESN'T KNOW, INPUT DK] [IF NO ANSWER, INPUT 999]

____

Logic: Show/hide trigger exists. Hidden unless: Question "Have you started your MP?" #40 is one of the following answers ("Yes - *This will enable more questions below.*")

Did you know about MP before it happened to you?*
( ) Yes (trigger)
( ) No
( ) DON'T KNOW/NO ANSWER

Logic: Hidden unless: Question "Did you know about MP before it happened to you?" #42 is one of the following answers ("Yes (trigger)")

Who first told you about it?*
[IF GIRL SAYS SCHOOL. ASK QUESTIONS TO DELINEATE PLAN/STRAIGHT TALK VS. ANOTHER SCHOOL PROGRAM].
( ) Mother
( ) Girl own age
( ) Aunt/Grandmother
( ) School/School program
( ) PLAN/straight talk education program
( ) Other (specify): ____*
( ) DON'T KNOW/NO ANSWER

Logic: Hidden unless: Question "Have you started your MP?" #40 is one of the following answers ("Yes - *This will enable more questions below.*")

Did you experience ANY of the following during your last MP? [READ]
[CHECK ALL THAT APPLY]*

[ ] Skin irritation / rashes in the pelvic area
[ ] Fear of panty soiling
[ ] Odour
[ ] Outside garment soiling
[ ] Embarrassment
[ ] Irritability/moodiness/depression/sadness
[ ] Insecurity
[ ] Fear that sanitary protection would fall out of underwear
[ ] [DO NOT READ] DON'T KNOW/NO ANSWER

Sanitary Protection Method

Logic: Show/hide trigger exists.

Page 6 - [RA: INTERVIEW DISPOSITION]*

( ) Consent obtained and MP
( ) Consent obtained but <u>NO MP</u>
( ) No consent

Logic: Show/hide trigger exists. Hidden unless: Question "Page 6 - [RA: INTERVIEW DISPOSITION]" is one of the following answers ("Consent obtained and MP")

Since the beginning of this school year, what do you USUALLY use to catch/absorb your MP? [*This question will enable more questions below.*]
[IF GIRL STATES MORE THAN ONE ASK, WHAT DO YOU MOST FREQUENTLY USE?]*

( ) AFRIPAD
( ) Cloth [VERIFY GIRL MEANS PIECES OF CLOTH NOT AFRIPAD]
( ) Toilet roll
( ) Sanitary pad
( ) Other (specify): ____*
( ) DON'T KNOW/NO ANSWER

Logic: Show/hide trigger exists. Hidden unless: Question "Since the beginning of this school year, what do you USUALLY use to catch/absorb your MP? [*This question will enable more questions below.*]
[IF GIRL STATES MORE THAN ONE ASK, WHAT DO YOU MOST FREQUENTLY USE?]" #45 is one of the following answers ("Cloth [VERIFY GIRL MEANS PIECES OF CLOTH NOT AFRIPAD]")

Who USUALLY provides you with the CLOTH that you use during your MP?
[IF GIRL STATES MORE THAN ONE ASK, WHO MOST FREQUENTLY PROVIDES YOU WITH THE CLOTH?]*

( ) Obtain it myself (trigger)
( ) Mother (trigger)
( ) Other family member (trigger)
( ) Non-related household member (trigger)
( ) Friend (trigger)
( ) Boyfriend (trigger)

( ) Teacher (trigger)
( ) Other (specify) (trigger): ____*
( ) DON'T KNOW/NO ANSWER

**Logic: Hidden unless: Question "Who USUALLY provides you with the CLOTH that you use during your MP?
[IF GIRL STATES MORE THAN ONE ASK, WHO MOST FREQUENTLY PROVIDES YOU WITH THE CLOTH?]" #46 is one of the following answers ("Mother (trigger)","Other family member (trigger)","Non-related household member (trigger)","Friend (trigger)","Boyfriend (trigger)","Teacher (trigger)","Other (specify) (trigger)")**

Where does this person USUALLY get the CLOTH?*
( ) Old cloth
( ) Shop
( ) Market
( ) School
( ) Rubbish
( ) From other females
( ) Other (specify): ____*
( ) DON'T KNOW/NO ANSWER

**Logic: Hidden unless: Question "Who USUALLY provides you with the CLOTH that you use during your MP?
[IF GIRL STATES MORE THAN ONE ASK, WHO MOST FREQUENTLY PROVIDES YOU WITH THE CLOTH?]" #46 is one of the following answers ("Obtain it myself (trigger)")**

Where do you USUALLY get the CLOTH you use when you're on your MP?*
( ) Old cloth
( ) Shop
( ) Market
( ) School
( ) Rubbish
( ) From other females
( ) *I reuse material.*
( ) Other (specify): ____*
( ) DON'T KNOW/NO ANSWER

**Logic: Hidden unless: Question "Since the beginning of this school year, what do you USUALLY use to catch/absorb your MP? [*This question will enable more questions below.*]
[IF GIRL STATES MORE THAN ONE ASK, WHAT DO YOU MOST FREQUENTLY USE?]" #45 is one of the following answers ("Cloth [VERIFY GIRL MEANS PIECES OF CLOTH NOT AFRIPAD]")**

[CLOTH] How many pieces do you have?*
[ENTER NUMBER] [IF GIRL DOES NOT KNOW, ENTER DK] [IF NO ANSWER, ENTER 999]
____

**Logic: Hidden unless: Question "Since the beginning of this school year, what do you USUALLY use to catch/absorb your MP? [*This question will enable more questions below.*]
[IF GIRL STATES MORE THAN ONE ASK, WHAT DO YOU MOST FREQUENTLY USE?]" #45 is one of the following answers ("Cloth [VERIFY GIRL MEANS PIECES OF CLOTH NOT AFRIPAD]")**

[CLOTH] How many do you use a day?*
[ENTER NUMBER] [IF GIRL DOES NOT KNOW, ENTER DK] [IF NO ANSWER, ENTER 999]

Logic: Hidden unless: Question "Since the beginning of this school year, what do you USUALLY use to catch/absorb your MP? [*This question will enable more questions below.*] [IF GIRL STATES MORE THAN ONE ASK, WHAT DO YOU MOST FREQUENTLY USE?]" #45 is one of the following answers ("Cloth [VERIFY GIRL MEANS PIECES OF CLOTH NOT AFRIPAD]")

[CLOTH] Do you share cloth with anyone else in your household?*
( ) Yes
( ) No
( ) DON'T KNOW/NO ANSWER

Logic: Hidden unless: Question "Since the beginning of this school year, what do you USUALLY use to catch/absorb your MP? [*This question will enable more questions below.*] [IF GIRL STATES MORE THAN ONE ASK, WHAT DO YOU MOST FREQUENTLY USE?]" #45 is one of the following answers ("Cloth [VERIFY GIRL MEANS PIECES OF CLOTH NOT AFRIPAD]")

[CLOTH] When you're on your MP, do you think you can rely on this method to keep you from soiling when you have to:
Walk a long distance*
( ) Yes
( ) No
( ) NOT APPLICABLE
( ) DON'T KNOW/NO ANSWER

Logic: Hidden unless: Question "Since the beginning of this school year, what do you USUALLY use to catch/absorb your MP? [*This question will enable more questions below.*] [IF GIRL STATES MORE THAN ONE ASK, WHAT DO YOU MOST FREQUENTLY USE?]" #45 is one of the following answers ("Cloth [VERIFY GIRL MEANS PIECES OF CLOTH NOT AFRIPAD]")

[CLOTH] When you're on your MP, do you think you can rely on this method to keep you from soiling when you have to:
Be gone from home for a long time*
( ) Yes
( ) No
( ) NOT APPLICABLE
( ) DON'T KNOW/NO ANSWER

Logic: Hidden unless: Question "Since the beginning of this school year, what do you USUALLY use to catch/absorb your MP? [*This question will enable more questions below.*] [IF GIRL STATES MORE THAN ONE ASK, WHAT DO YOU MOST FREQUENTLY USE?]" #45 is one of the following answers ("Cloth [VERIFY GIRL MEANS PIECES OF CLOTH NOT AFRIPAD]")

[CLOTH] When you're on your MP, do you think you can rely on this method to keep you from soiling when you have to:
Move quickly or strenuously*
( ) Yes
( ) No
( ) NOT APPLICABLE
( ) DON'T KNOW/NO ANSWER

Logic: Hidden unless: Question "Since the beginning of this school year, what do you USUALLY use to catch/absorb your MP? [*This question will enable more questions below.*] [IF GIRL STATES MORE THAN ONE ASK, WHAT DO YOU MOST FREQUENTLY USE?]" #45 is one of the following answers ("Cloth [VERIFY GIRL MEANS PIECES OF CLOTH NOT AFRIPAD]")

[CLOTH] When you're on your MP, do you think you can rely on this method to keep you from soiling when you have to:
Sit for a long time*

( ) Yes
( ) No
( ) NOT APPLICABLE
( ) DON'T KNOW/NO ANSWER

Logic: Hidden unless: Question "Since the beginning of this school year, what do you USUALLY use to catch/absorb your MP? [*This question will enable more questions below.*] [IF GIRL STATES MORE THAN ONE ASK, WHAT DO YOU MOST FREQUENTLY USE?]" #45 is one of the following answers ("Cloth [VERIFY GIRL MEANS PIECES OF CLOTH NOT AFRIPAD]")

[CLOTH] When you're on your MP, do you think you can rely on this method to keep you from soiling when you have to:
Be around males*

( ) Yes
( ) No
( ) NOT APPLICABLE
( ) DON'T KNOW/NO ANSWER

Logic: Hidden unless: Question "Since the beginning of this school year, what do you USUALLY use to catch/absorb your MP? [*This question will enable more questions below.*] [IF GIRL STATES MORE THAN ONE ASK, WHAT DO YOU MOST FREQUENTLY USE?]" #45 is one of the following answers ("Cloth [VERIFY GIRL MEANS PIECES OF CLOTH NOT AFRIPAD]")

[CLOTH] When you're on your MP, do you think you can rely on this method to keep you from soiling when you have to:
Go to the farm away from home*

( ) Yes
( ) No
( ) NOT APPLICABLE
( ) DON'T KNOW/NO ANSWER

Logic: Hidden unless: Question "Since the beginning of this school year, what do you USUALLY use to catch/absorb your MP? [*This question will enable more questions below.*] [IF GIRL STATES MORE THAN ONE ASK, WHAT DO YOU MOST FREQUENTLY USE?]" #45 is one of the following answers ("Cloth [VERIFY GIRL MEANS PIECES OF CLOTH NOT AFRIPAD]")

[CLOTH] When you're on your MP, do you think you can rely on this method to keep you from soiling when you have to:
Go to school*

( ) Yes
( ) No
( ) NOT APPLICABLE
( ) DON'T KNOW/NO ANSWER

**Logic: Hidden unless: Question "Since the beginning of this school year, what do you USUALLY use to catch/absorb your MP? [*This question will enable more questions below.*]
[IF GIRL STATES MORE THAN ONE ASK, WHAT DO YOU MOST FREQUENTLY USE?]" #45 is one of the following answers ("Cloth [VERIFY GIRL MEANS PIECES OF CLOTH NOT AFRIPAD]")**

[CLOTH] When you're on your MP, do you think you can rely on this method to keep you from soiling when you have to:
Go to the market*

( ) Yes
( ) No
( ) NOT APPLICABLE
( ) DON'T KNOW/NO ANSWER

**Logic: Hidden unless: Question "Since the beginning of this school year, what do you USUALLY use to catch/absorb your MP? [*This question will enable more questions below.*]
[IF GIRL STATES MORE THAN ONE ASK, WHAT DO YOU MOST FREQUENTLY USE?]" #45 is one of the following answers ("Cloth [VERIFY GIRL MEANS PIECES OF CLOTH NOT AFRIPAD]")**

How much of a problem is leaking while using CLOTH? Is it...[READ]*

( ) Not a problem at all,
( ) A little bit of a problem, or
( ) A big problem?
( ) [DO NOT READ] DON'T KNOW/NO ANSWER

**Logic: Hidden unless: Question "Since the beginning of this school year, what do you USUALLY use to catch/absorb your MP? [*This question will enable more questions below.*]
[IF GIRL STATES MORE THAN ONE ASK, WHAT DO YOU MOST FREQUENTLY USE?]" #45 is one of the following answers ("Cloth [VERIFY GIRL MEANS PIECES OF CLOTH NOT AFRIPAD]")**

Does ANYONE else in your household use cloth?*

( ) Yes
( ) No
( ) DON'T KNOW/NO ANSWER

**Logic: Show/hide trigger exists. Hidden unless: Question "Since the beginning of this school year, what do you USUALLY use to catch/absorb your MP? [*This question will enable more questions below.*]
[IF GIRL STATES MORE THAN ONE ASK, WHAT DO YOU MOST FREQUENTLY USE?]" #45 is one of the following answers ("Cloth [VERIFY GIRL MEANS PIECES OF CLOTH NOT AFRIPAD]")**

Did you ever try to wash the CLOTH?*

( ) Yes (trigger)
( ) No (trigger)
( ) DON'T KNOW/NO ANSWER

Logic: Hidden unless: Question "Did you ever try to wash the CLOTH?" #62 is one of the following answers ("No (trigger)")

Why didn't you try to wash the CLOTH?
[CHECK ALL THAT APPLY]*

[ ] Had trouble getting water
[ ] Could not afford soap

[ ] Could afford soap but just didn't have any
[ ] Did not have enough time
[ ] Decided not to use it again
[ ] Did not have enough privacy
[ ] It was disgusting
[ ] Other (specify): ____*
[ ] DON'T KNOW/NO ANSWER

Logic: Show/hide trigger exists. Hidden unless: Question "Did you ever try to wash the CLOTH?" #62 is one of the following answers ("Yes (trigger)")

Did you use soap...[READ]*
( ) Every time,
( ) Sometimes, or
( ) Never?
( ) [DO NOT READ] DON'T KNOW/NO ANSWER

Logic: Hidden unless: Question "Did you use soap...[READ]" #64 is one of the following answers ("Sometimes, or","Never?")

Why didn't you use soap?
[CHECK ALL THAT APPLY]*
[ ] Often don't have soap
[ ] Don't need soap to get it clean
[ ] Don't care to use soap
[ ] Other (specify): ____*
[ ] DON'T KNOW/NO ANSWER

Logic: Hidden unless: Question "Did you ever try to wash the CLOTH?" #62 is one of the following answers ("Yes (trigger)")

Did you use hot water or cold water?*
( ) Hot water
( ) Cold water
( ) DON'T KNOW/NO ANSWER

Logic: Hidden unless: Question "Did you ever try to wash the CLOTH?" #62 is one of the following answers ("Yes (trigger)")

Was the water clean or dirty?*
( ) Clean
( ) Dirty
( ) DON'T KNOW/NO ANSWER

Logic: Hidden unless: Question "Did you ever try to wash the CLOTH?" #62 is one of the following answers ("Yes (trigger)")

Did you feel disgusted by washing the CLOTH?*
( ) Yes
( ) No
( ) DON'T KNOW/NO ANSWER

Logic: Hidden unless: Question "Did you ever try to wash the CLOTH?" #62 is one of the following answers ("Yes (trigger)")

Did you worry about being observed washing the CLOTH?*

( ) Yes
( ) No
( ) DON'T KNOW/NO ANSWER

Logic: Show/hide trigger exists. Hidden unless: Question "Did you ever try to wash the CLOTH?" #62 is one of the following answers ("Yes (trigger)")

Did you try to dry the CLOTH?*
( ) Yes (trigger)
( ) No (trigger)
( ) DON'T KNOW/NO ANSWER

Logic: Hidden unless: Question "Did you try to dry the CLOTH?" #70 is one of the following answers ("No (trigger)")

Why didn't you try to dry the CLOTH?
[CHECK ALL THAT APPLY]*
[ ] Did not have enough time
[ ] Decided not to use it again
[ ] Did not have enough privacy
[ ] It was disgusting
[ ] Other (specify): ____*
[ ] DON'T KNOW/NO ANSWER

Logic: Hidden unless: Question "Did you try to dry the CLOTH?" #70 is one of the following answers ("Yes (trigger)")

Did you worry about drying the CLOTH?*
( ) Yes
( ) No
( ) DON'T KNOW/NO ANSWER

Logic: Hidden unless: Question "Did you try to dry the CLOTH?" #70 is one of the following answers ("Yes (trigger)")

Where did you hang the CLOTH to dry?
[CHECK ALL THAT APPLY]*
[ ] Dried pad under bed
[ ] Dried pad outdoors
[ ] Dried pad in dorm where other girls could see, such as on a peg
[ ] Dried pad in another secret place
[ ] Other (specify): ____*
[ ] DON'T KNOW/NO ANSWER

Logic: Hidden unless: Question "Did you try to dry the CLOTH?" #70 is one of the following answers ("Yes (trigger)")

How many hours did it take to dry the CLOTH completely?*
[ENTER NUMBER] [IF GIRL DOES NOT KNOW, ENTER DK] [IF NO ANSWER, ENTER 999]
____

Logic: Hidden unless: Question "Did you ever try to wash the CLOTH?" #62 is one of the following answers ("Yes (trigger)")

How often did you wear the CLOTH damp? Would you say...[READ]*
( ) Usually

( ) Sometimes, or
( ) Never?
( ) [DO NOT READ] DON'T KNOW/NO ANSWER

Logic: Show/hide trigger exists. Hidden unless: Question "Since the beginning of this school year, what do you USUALLY use to catch/absorb your MP? [*This question will enable more questions below.*]
[IF GIRL STATES MORE THAN ONE ASK, WHAT DO YOU MOST FREQUENTLY USE?]" #45 is one of the following answers ("Toilet roll")

Who USUALLY provides you with the TOILET ROLL that you use during your MP?
[IF GIRL STATES MORE THAN ONE ASK, WHO MOST FREQUENTLY PROVIDES YOU THE TOILET ROLL?]*
( ) Obtain it myself (trigger)
( ) Mother (trigger)
( ) Other family member (trigger)
( ) Non-related household member (trigger)
( ) Friend (trigger)
( ) Boyfriend (trigger)
( ) Teacher (trigger)
( ) Other (specify) (trigger): ____*
( ) DON'T KNOW/NO ANSWER

Logic: Hidden unless: Question "Who USUALLY provides you with the TOILET ROLL that you use during your MP?
[IF GIRL STATES MORE THAN ONE ASK, WHO MOST FREQUENTLY PROVIDES YOU THE TOILET ROLL?]" #76 is one of the following answers ("Mother (trigger)","Other family member (trigger)","Non-related household member (trigger)","Friend (trigger)","Boyfriend (trigger)","Teacher (trigger)","Other (specify) (trigger)")

Where does this person USUALLY get the TOILET ROLL?*
( ) Shop
( ) Market
( ) School
( ) Rubbish
( ) From other females
( ) Other (specify): ____*
( ) DON'T KNOW/NO ANSWER

Logic: Hidden unless: Question "Who USUALLY provides you with the TOILET ROLL that you use during your MP?
[IF GIRL STATES MORE THAN ONE ASK, WHO MOST FREQUENTLY PROVIDES YOU THE TOILET ROLL?]" #76 is one of the following answers ("Obtain it myself (trigger)")

Where do you USUALLY get the TOILET ROLL you use when you're on your MP?*
( ) Shop
( ) Market
( ) School
( ) Rubbish
( ) From other females
( ) Other (specify): ____*
( ) DON'T KNOW/NO ANSWER

Logic: Hidden unless: Question "Since the beginning of this school year, what do you USUALLY use to catch/absorb your MP? [*This question will enable more questions below.*]

[IF GIRL STATES MORE THAN ONE ASK, WHAT DO YOU MOST FREQUENTLY USE?]" #45 is one of the following answers ("Toilet roll")

[TOILET ROLL] When you're on your MP, do you think you can rely on this method to keep you from soiling when you have to:

Walk a long distance

*****
( ) Yes
( ) No
( ) NOT APPLICABLE
( ) DON'T KNOW/NO ANSWER

Logic: Hidden unless: Question "Since the beginning of this school year, what do you USUALLY use to catch/absorb your MP? [*This question will enable more questions below.*] [IF GIRL STATES MORE THAN ONE ASK, WHAT DO YOU MOST FREQUENTLY USE?]" #45 is one of the following answers ("Toilet roll")

[TOILET ROLL] When you're on your MP, do you think you can rely on this method to keep you from soiling when you have to:

Be gone from home for a long time

*****
( ) Yes
( ) No
( ) NOT APPLICABLE
( ) DON'T KNOW/NO ANSWER

Logic: Hidden unless: Question "Since the beginning of this school year, what do you USUALLY use to catch/absorb your MP? [*This question will enable more questions below.*] [IF GIRL STATES MORE THAN ONE ASK, WHAT DO YOU MOST FREQUENTLY USE?]" #45 is one of the following answers ("Toilet roll")

[TOILET ROLL] When you're on your MP, do you think you can rely on this method to keep you from soiling when you have to:
Move quickly or strenuously*
( ) Yes
( ) No
( ) NOT APPLICABLE
( ) DON'T KNOW/NO ANSWER

Logic: Hidden unless: Question "Since the beginning of this school year, what do you USUALLY use to catch/absorb your MP? [*This question will enable more questions below.*] [IF GIRL STATES MORE THAN ONE ASK, WHAT DO YOU MOST FREQUENTLY USE?]" #45 is one of the following answers ("Toilet roll")

[TOILET ROLL] When you're on your MP, do you think you can rely on this method to keep you from soiling when you have to:
Sit for a long time*
( ) Yes
( ) No
( ) NOT APPLICABLE
( ) DON'T KNOW/NO ANSWER

Logic: Hidden unless: Question "Since the beginning of this school year, what do you USUALLY use to catch/absorb your MP? [*This question will enable more questions below.*]

[IF GIRL STATES MORE THAN ONE ASK, WHAT DO YOU MOST FREQUENTLY USE?]" #45 is one of the following answers ("Toilet roll")

[TOILET ROLL] When you're on your MP, do you think you can rely on this method to keep you from soiling when you have to:
Be around males*

( ) Yes
( ) No
( ) NOT APPLICABLE
( ) DON'T KNOW/NO ANSWER

Logic: Hidden unless: Question "Since the beginning of this school year, what do you USUALLY use to catch/absorb your MP? [*This question will enable more questions below.*] [IF GIRL STATES MORE THAN ONE ASK, WHAT DO YOU MOST FREQUENTLY USE?]" #45 is one of the following answers ("Toilet roll")

[TOILET ROLL] When you're on your MP, do you think you can rely on this method to keep you from soiling when you have to:
Go to the farm away from home*

( ) Yes
( ) No
( ) NOT APPLICABLE
( ) DON'T KNOW/NO ANSWER

Logic: Hidden unless: Question "Since the beginning of this school year, what do you USUALLY use to catch/absorb your MP? [*This question will enable more questions below.*] [IF GIRL STATES MORE THAN ONE ASK, WHAT DO YOU MOST FREQUENTLY USE?]" #45 is one of the following answers ("Toilet roll")

[TOILET ROLL] When you're on your MP, do you think you can rely on this method to keep you from soiling when you have to:
Go to school*

( ) Yes
( ) No
( ) NOT APPLICABLE
( ) DON'T KNOW/NO ANSWER

Logic: Hidden unless: Question "Since the beginning of this school year, what do you USUALLY use to catch/absorb your MP? [*This question will enable more questions below.*] [IF GIRL STATES MORE THAN ONE ASK, WHAT DO YOU MOST FREQUENTLY USE?]" #45 is one of the following answers ("Toilet roll")

[TOILET ROLL] When you're on your MP, do you think you can rely on this method to keep you from soiling when you have to:
Go to the market*

( ) Yes
( ) No
( ) NOT APPLICABLE
( ) DON'T KNOW/NO ANSWER

Logic: Hidden unless: Question "Since the beginning of this school year, what do you USUALLY use to catch/absorb your MP? [*This question will enable more questions below.*] [IF GIRL STATES MORE THAN ONE ASK, WHAT DO YOU MOST FREQUENTLY USE?]" #45 is one of the following answers ("Toilet roll")

How much of a problem is leaking while using TOILET ROLL? Is it...[READ]*

( ) Not a problem at all,
( ) A little bit of a problem, or
( ) A big problem?
( ) [DO NOT READ] DON'T KNOW/NO ANSWER

Logic: Hidden unless: Question "Since the beginning of this school year, what do you USUALLY use to catch/absorb your MP? [*This question will enable more questions below.*] [IF GIRL STATES MORE THAN ONE ASK, WHAT DO YOU MOST FREQUENTLY USE?]" #45 is one of the following answers ("Toilet roll")

Does anyone else in your household use toilet roll?*
( ) Yes
( ) No
( ) DON'T KNOW/NO ANSWER

**Logic: Show/hide trigger exists. Hidden unless: Question "Since the beginning of this school year, what do you USUALLY use to catch/absorb your MP? [*This question will enable more questions below.*]
[IF GIRL STATES MORE THAN ONE ASK, WHAT DO YOU MOST FREQUENTLY USE?]" #45 is one of the following answers ("Sanitary pad")**

Who USUALLY provides you with the SANITARY PADS that you use during your MP? [IF GIRL STATES MORE THAN ONE ASK, WHO MOST FREQUENTLY PROVIDES YOU THE SANITARY PAD?]*
( ) Obtain it myself (trigger)
( ) Mother (trigger)
( ) Other family member (trigger)
( ) Non-related household member (trigger)
( ) Friend (trigger)
( ) Boyfriend (trigger)
( ) Teacher (trigger)
( ) Other (specify) (trigger): ____*
( ) DON'T KNOW/NO ANSWER

**Logic: Hidden unless: Question "Who USUALLY provides you with the SANITARY PADS that you use during your MP?
[IF GIRL STATES MORE THAN ONE ASK, WHO MOST FREQUENTLY PROVIDES YOU THE SANITARY PAD?]" #89 is one of the following answers ("Mother (trigger)","Other family member (trigger)","Non-related household member (trigger)","Friend (trigger)","Boyfriend (trigger)","Teacher (trigger)","Other (specify) (trigger)")**

Where does this person USUALLY get the SANITARY PADS?*
( ) Shop
( ) Market
( ) School
( ) Rubbish
( ) From other females
( ) Other (specify): ____*
( ) DON'T KNOW/NO ANSWER

**Logic: Hidden unless: Question "Who USUALLY provides you with the SANITARY PADS that you use during your MP?
[IF GIRL STATES MORE THAN ONE ASK, WHO MOST FREQUENTLY PROVIDES YOU THE SANITARY PAD?]" #89 is one of the following answers ("Obtain it myself (trigger)")**

Where do you USUALLY get the SANITARY PADS?*

( ) Shop
( ) Market
( ) School
( ) Rubbish
( ) From other females
( ) Other (specify): ____*
( ) DON'T KNOW/NO ANSWER

Logic: Hidden unless: Question "Since the beginning of this school year, what do you USUALLY use to catch/absorb your MP? [*This question will enable more questions below.*] [IF GIRL STATES MORE THAN ONE ASK, WHAT DO YOU MOST FREQUENTLY USE?]" #45 is one of the following answers ("Sanitary pad")

[SANITARY PAD] When you're on your MP, do you think you can rely on this method to keep you from soiling when you have to:
Walk a long distance*
( ) Yes
( ) No
( ) NOT APPLICABLE
( ) DON'T KNOW/NO ANSWER

Logic: Hidden unless: Question "Since the beginning of this school year, what do you USUALLY use to catch/absorb your MP? [*This question will enable more questions below.*] [IF GIRL STATES MORE THAN ONE ASK, WHAT DO YOU MOST FREQUENTLY USE?]" #45 is one of the following answers ("Sanitary pad")

[SANITARY PAD] When you're on your MP, do you think you can rely on this method to keep you from soiling when you have to:
Be gone from home for a long time*
( ) Yes
( ) No
( ) NOT APPLICABLE
( ) DON'T KNOW/NO ANSWER

Logic: Hidden unless: Question "Since the beginning of this school year, what do you USUALLY use to catch/absorb your MP? [*This question will enable more questions below.*] [IF GIRL STATES MORE THAN ONE ASK, WHAT DO YOU MOST FREQUENTLY USE?]" #45 is one of the following answers ("Sanitary pad")

[SANITARY PAD] When you're on your MP, do you think you can rely on this method to keep you from soiling when you have to:
Move quickly or strenuously*
( ) Yes
( ) No
( ) NOT APPLICABLE
( ) DON'T KNOW/NO ANSWER

Logic: Hidden unless: Question "Since the beginning of this school year, what do you USUALLY use to catch/absorb your MP? [*This question will enable more questions below.*] [IF GIRL STATES MORE THAN ONE ASK, WHAT DO YOU MOST FREQUENTLY USE?]" #45 is one of the following answers ("Sanitary pad")

[SANITARY PAD] When you're on your MP, do you think you can rely on this method to keep you from soiling when you have to:
Sit for a long time*
( ) Yes

( ) No
( ) NOT APPLICABLE
( ) DON'T KNOW/NO ANSWER

Logic: Hidden unless: Question "Since the beginning of this school year, what do you USUALLY use to catch/absorb your MP? [*This question will enable more questions below.*] [IF GIRL STATES MORE THAN ONE ASK, WHAT DO YOU MOST FREQUENTLY USE?]" #45 is one of the following answers ("Sanitary pad")

[SANITARY PAD] When you're on your MP, do you think you can rely on this method to keep you from soiling when you have to:
Be around males*
( ) Yes
( ) No
( ) NOT APPLICABLE
( ) DON'T KNOW/NO ANSWER

Logic: Hidden unless: Question "Since the beginning of this school year, what do you USUALLY use to catch/absorb your MP? [*This question will enable more questions below.*] [IF GIRL STATES MORE THAN ONE ASK, WHAT DO YOU MOST FREQUENTLY USE?]" #45 is one of the following answers ("Sanitary pad")

[SANITARY PAD] When you're on your MP, do you think you can rely on this method to keep you from soiling when you have to:
Go to the farm away from home*
( ) Yes
( ) No
( ) NOT APPLICABLE
( ) DON'T KNOW/NO ANSWER

Logic: Hidden unless: Question "Since the beginning of this school year, what do you USUALLY use to catch/absorb your MP? [*This question will enable more questions below.*] [IF GIRL STATES MORE THAN ONE ASK, WHAT DO YOU MOST FREQUENTLY USE?]" #45 is one of the following answers ("Sanitary pad")

[SANITARY PAD] When you're on your MP, do you think you can rely on this method to keep you from soiling when you have to:
Go to school*
( ) Yes
( ) No
( ) NOT APPLICABLE
( ) DON'T KNOW/NO ANSWER

Logic: Hidden unless: Question "Since the beginning of this school year, what do you USUALLY use to catch/absorb your MP? [*This question will enable more questions below.*] [IF GIRL STATES MORE THAN ONE ASK, WHAT DO YOU MOST FREQUENTLY USE?]" #45 is one of the following answers ("Sanitary pad")

[SANITARY PAD] When you're on your MP, do you think you can rely on this method to keep you from soiling when you have to:
Go to the market*
( ) Yes
( ) No
( ) NOT APPLICABLE
( ) DON'T KNOW/NO ANSWER

**Logic: Hidden unless: Question "Since the beginning of this school year, what do you USUALLY use to catch/absorb your MP? [*This question will enable more questions below.*]
[IF GIRL STATES MORE THAN ONE ASK, WHAT DO YOU MOST FREQUENTLY USE?]" #45 is one of the following answers ("Sanitary pad")**

How much of a problem is leaking while using SANITARY PAD? Is it...[READ]*
( ) Not a problem at all,
( ) A little bit of a problem, or
( ) A big problem?
( ) [DO NOT READ] DON'T KNOW/NO ANSWER

**Logic: Hidden unless: Question "Since the beginning of this school year, what do you USUALLY use to catch/absorb your MP? [*This question will enable more questions below.*]
[IF GIRL STATES MORE THAN ONE ASK, WHAT DO YOU MOST FREQUENTLY USE?]" #45 is one of the following answers ("Sanitary pad")**

Does anyone else in your household use sanitary pads?*
( ) Yes
( ) No
( ) DON'T KNOW/NO ANSWER

**Logic: Show/hide trigger exists. Hidden unless: Question "Since the beginning of this school year, what do you USUALLY use to catch/absorb your MP? [*This question will enable more questions below.*]
[IF GIRL STATES MORE THAN ONE ASK, WHAT DO YOU MOST FREQUENTLY USE?]" #45 is one of the following answers ("Other (specify)")**

**[OTHER] Who USUALLY provides you with the material that you use during your MP?
[IF GIRL STATES MORE THAN ONE ASK, WHO MOST FREQUENTLY PROVIDES YOU WITH THE MATERIAL?]***
( ) Obtain it myself (trigger)
( ) Mother (trigger)
( ) Other family member (trigger)
( ) Non-related household member (trigger)
( ) Friend (trigger)
( ) Boyfriend (trigger)
( ) Teacher (trigger)
( ) Other (specify) (trigger): ____*
( ) DON'T KNOW/NO ANSWER

**Logic: Hidden unless: Question "[OTHER] Who USUALLY provides you with the material that you use during your MP?
[IF GIRL STATES MORE THAN ONE ASK, WHO MOST FREQUENTLY PROVIDES YOU WITH THE MATERIAL?]" #102 is one of the following answers ("Mother (trigger)","Other family member (trigger)","Non-related household member (trigger)","Friend (trigger)","Boyfriend (trigger)","Teacher (trigger)","Other (specify) (trigger)")**

[OTHER] Where does this person USUALLY get the material?*
( ) Old cloth
( ) Shop
( ) Market
( ) School
( ) Rubbish
( ) From other females
( ) Other (specify): ____*
( ) DON'T KNOW/NO ANSWER

**Logic: Hidden unless: Question "[OTHER] Who USUALLY provides you with the material that you use during your MP?
[IF GIRL STATES MORE THAN ONE ASK, WHO MOST FREQUENTLY PROVIDES YOU WITH THE MATERIAL?]" #102 is one of the following answers ("Obtain it myself (trigger)")**

[OTHER] Where do you USUALLY get the material you use when you're on your MP?*
( ) Old cloth
( ) Shop
( ) Market
( ) School
( ) Rubbish
( ) From other females
( ) *I reuse material.*
( ) Other (specify): ____*
( ) DON'T KNOW/NO ANSWER

**Logic: Hidden unless: Question "Since the beginning of this school year, what do you USUALLY use to catch/absorb your MP? [*This question will enable more questions below.*]
[IF GIRL STATES MORE THAN ONE ASK, WHAT DO YOU MOST FREQUENTLY USE?]" #45 is one of the following answers ("Other (specify)")**

**[OTHER] When you're on your MP, do you think you can rely on this method to keep you from soiling when you have to:
Walk a long distance***
( ) Yes
( ) No
( ) NOT APPLICABLE
( ) DON'T KNOW/NO ANSWER

**Logic: Hidden unless: Question "Since the beginning of this school year, what do you USUALLY use to catch/absorb your MP? [*This question will enable more questions below.*]
[IF GIRL STATES MORE THAN ONE ASK, WHAT DO YOU MOST FREQUENTLY USE?]" #45 is one of the following answers ("Other (specify)")**

**[OTHER] When you're on your MP, do you think you can rely on this method to keep you from soiling when you have to:
Be gone from home for a long time***
( ) Yes
( ) No
( ) NOT APPLICABLE
( ) DON'T KNOW/NO ANSWER

**Logic: Hidden unless: Question "Since the beginning of this school year, what do you USUALLY use to catch/absorb your MP? [*This question will enable more questions below.*]
[IF GIRL STATES MORE THAN ONE ASK, WHAT DO YOU MOST FREQUENTLY USE?]" #45 is one of the following answers ("Other (specify)")**

**[OTHER] When you're on your MP, do you think you can rely on this method to keep you from soiling when you have to:
Move quickly or strenuously***
( ) Yes
( ) No
( ) NOT APPLICABLE
( ) DON'T KNOW/NO ANSWER

Logic: Hidden unless: Question "Since the beginning of this school year, what do you USUALLY use to catch/absorb your MP? [*This question will enable more questions below.*] [IF GIRL STATES MORE THAN ONE ASK, WHAT DO YOU MOST FREQUENTLY USE?]" #45 is one of the following answers ("Other (specify)")

[OTHER] When you're on your MP, do you think you can rely on this method to keep you from soiling when you have to:
Sit for a long time*
( ) Yes
( ) No
( ) NOT APPLICABLE
( ) DON'T KNOW/NO ANSWER

Logic: Hidden unless: Question "Since the beginning of this school year, what do you USUALLY use to catch/absorb your MP? [*This question will enable more questions below.*] [IF GIRL STATES MORE THAN ONE ASK, WHAT DO YOU MOST FREQUENTLY USE?]" #45 is one of the following answers ("Other (specify)")

[OTHER] When you're on your MP, do you think you can rely on this method to keep you from soiling when you have to:
Be around males*
( ) Yes
( ) No
( ) NOT APPLICABLE
( ) DON'T KNOW/NO ANSWER

Logic: Hidden unless: Question "Since the beginning of this school year, what do you USUALLY use to catch/absorb your MP? [*This question will enable more questions below.*] [IF GIRL STATES MORE THAN ONE ASK, WHAT DO YOU MOST FREQUENTLY USE?]" #45 is one of the following answers ("Other (specify)")

[OTHER] When you're on your MP, do you think you can rely on this method to keep you from soiling when you have to:
Go to the farm away from home*
( ) Yes
( ) No
( ) NOT APPLICABLE
( ) DON'T KNOW/NO ANSWER

Logic: Hidden unless: Question "Since the beginning of this school year, what do you USUALLY use to catch/absorb your MP? [*This question will enable more questions below.*] [IF GIRL STATES MORE THAN ONE ASK, WHAT DO YOU MOST FREQUENTLY USE?]" #45 is one of the following answers ("Other (specify)")

[OTHER] When you're on your MP, do you think you can rely on this method to keep you from soiling when you have to:
Go to school*
( ) Yes
( ) No
( ) NOT APPLICABLE
( ) DON'T KNOW/NO ANSWER

Logic: Hidden unless: Question "Since the beginning of this school year, what do you USUALLY use to catch/absorb your MP? [*This question will enable more questions below.*]

[IF GIRL STATES MORE THAN ONE ASK, WHAT DO YOU MOST FREQUENTLY USE?]" #45 is one of the following answers ("Other (specify)")

[OTHER] When you're on your MP, do you think you can rely on this method to keep you from soiling when you have to:
Go to the market*
( ) Yes
( ) No
( ) NOT APPLICABLE
( ) DON'T KNOW/NO ANSWER

Logic: Hidden unless: Question "Since the beginning of this school year, what do you USUALLY use to catch/absorb your MP? [*This question will enable more questions below.*]
[IF GIRL STATES MORE THAN ONE ASK, WHAT DO YOU MOST FREQUENTLY USE?]" #45 is one of the following answers ("Other (specify)")

[OTHER] How much of a problem is leaking while using this material? Is it...[READ]*
( ) Not a problem at all,
( ) A little bit of a problem, or
( ) A big problem?
( ) [DO NOT READ] DON'T KNOW/NO ANSWER

Logic: Hidden unless: Question "Since the beginning of this school year, what do you USUALLY use to catch/absorb your MP? [*This question will enable more questions below.*]
[IF GIRL STATES MORE THAN ONE ASK, WHAT DO YOU MOST FREQUENTLY USE?]" #45 is one of the following answers ("Other (specify)")

[OTHER] Does anyone else in your household use this material?*
( ) Yes
( ) No
( ) DON'T KNOW/NO ANSWER

Logic: Hidden unless: Question "Since the beginning of this school year, what do you USUALLY use to catch/absorb your MP? [*This question will enable more questions below.*]
[IF GIRL STATES MORE THAN ONE ASK, WHAT DO YOU MOST FREQUENTLY USE?]" #45 is one of the following answers ("Cloth [VERIFY GIRL MEANS PIECES OF CLOTH NOT AFRIPAD]","Toilet roll","Sanitary pad","Other (specify)","DON'T KNOW/NO ANSWER")

Logic: Show/hide trigger exists. Hidden unless: Question "Since the beginning of this school year, what do you USUALLY use to catch/absorb your MP? [*This question will enable more questions below.*]
[IF GIRL STATES MORE THAN ONE ASK, WHAT DO YOU MOST FREQUENTLY USE?]" #45 is one of the following answers ("Cloth [VERIFY GIRL MEANS PIECES OF CLOTH NOT AFRIPAD]","Toilet roll","Sanitary pad","Other (specify)","DON'T KNOW/NO ANSWER")

Were you given a pack of AFRIPADs since we came last year?*
( ) Yes (trigger)
( ) No
( ) No answer

Logic: Show/hide trigger exists. Hidden unless: Question "Were you given a pack of AFRIPADs since we came last year?" #115 is one of the following answers ("Yes (trigger)")

Did you...[READ]*
( ) Never use them?
( ) Use them only once?
( ) Use them more than once and are still using them? Or
( ) Use them more than once but stop using them?
( ) [DO NOT READ] DON'T KNOW/NO ANSWER

Logic: Hidden unless: Question "Did you...[READ]" #116 is one of the following answers ("Never use them?")

Which of the following are reasons you decided not to try the AFRIPAD? Because...[READ] [CHECK ALL THAT APPLY]*
[ ] You had enough money for regular pads?
[ ] You trusted regular pads more?
[ ] Other girls told you the AFRIPADS were no good?
[ ] The AFRIPADs did not look like they would work well?
[ ] You were afraid the AFRIPAD would smell?
[ ] You were afraid the AFRIPAD would show through clothing?
[ ] You were afraid the AFRIPAD would leak?
[ ] You did not know how to dispose of the AFRIPAD?
[ ] You did not have any way to clean the AFRIPAD?
[ ] You did not have any way to dry the AFRIPAD?
[ ] You were too disgusted to wash your own blood?
[ ] [DO NOT READ] DON'T KNOW/NO ANSWER

Logic: Hidden unless: Question "Did you...[READ]" #116 is one of the following answers ("Never use them?")

Are there any other reasons you decided not to use the AFRIPAD?*
( ) Yes (specify): ____*
( ) No
( ) DON'T KNOW/NO ANSWER

Logic: Hidden unless: Question "Did you...[READ]" #116 is one of the following answers ("Use them only once?","Use them more than once but stop using them?")

Which of the following are reasons you decided to stop using the AFRIPAD? Because...[READ] [CHECK ALL THAT APPLY]*
[ ] You had enough money for regular pads?
[ ] You trusted regular pads more?
[ ] Other girls told you the AFRIPADS were no good?
[ ] The AFRIPADs did not work well?
[ ] The AFRIPADs smelled?
[ ] The AFRIPADs showed through clothing?
[ ] The AFRIPADs leaked?
[ ] You did not know how to dispose of the AFRIPAD?
[ ] You did not have any way to clean the AFRIPAD?
[ ] You did not have any way to dry the AFRIPAD?
[ ] You were too disgusted to wash your own blood?
[ ] [DO NOT READ] DON'T KNOW/NO ANSWER

Logic: Hidden unless: Question "Did you...[READ]" #116 is one of the following answers ("Use them only once?","Use them more than once but stop using them?")

Are there any other reasons you decided to stop using the AFRIPAD?*
( ) Yes (specify): ____*
( ) No
( ) DON'T KNOW/NO ANSWER

Logic: Hidden unless: Question "Were you given a pack of AFRIPADs since we came last year?" #115 is one of the following answers ("Yes (trigger)")

Did you sell any part of your AFRIPAD pack? Would you say...[READ]*
( ) Yes, the whole thing?
( ) Yes, part of the pack? Or
( ) No, none of it?
( ) [DO NOT READ] DON'T KNOW/NO ANSWER

Logic: Hidden unless: Question "Since the beginning of this school year, what do you USUALLY use to catch/absorb your MP? [*This question will enable more questions below.*] [IF GIRL STATES MORE THAN ONE ASK, WHAT DO YOU MOST FREQUENTLY USE?]" #45 is one of the following answers ("AFRIPAD")

Did you sell any part of your AFRIPAD pack? Would you say...[READ]*
( ) Yes, part of the pack? Or
( ) No, none of it?
( ) [DO NOT READ] DON'T KNOW/NO ANSWER

Logic: Show/hide trigger exists. Hidden unless: (Question "Did you sell any part of your AFRIPAD pack? Would you say...[READ]" #121 is one of the following answers ("Yes, part of the pack? Or","No, none of it?","[DO NOT READ] DON'T KNOW/NO ANSWER") OR Question "Did you sell any part of your AFRIPAD pack? Would you say...[READ]" #122 is one of the following answers ("Yes, part of the pack? Or","No, none of it?","[DO NOT READ] DON'T KNOW/NO ANSWER"))

Did you share any part of your AFRIPAD pack?*
( ) Yes (trigger)
( ) No
( ) DON'T KNOW/NO ANSWER

Logic: Hidden unless: Question "Did you share any part of your AFRIPAD pack?" #123 is one of the following answers ("Yes (trigger)")

Who did you share with?
[CHECK ALL THAT APPLY]*
[ ] Family
[ ] Friends
[ ] Other girls at school
[ ] Other (specify): ____*
[ ] DON'T KNOW/NO ANSWER

Logic: Hidden unless: (Question "Did you sell any part of your AFRIPAD pack? Would you say...[READ]" #121 is one of the following answers ("No, none of it?","[DO NOT READ] DON'T KNOW/NO ANSWER") AND Question "Did you share any part of your AFRIPAD pack?" #123 is one of the following answers ("No","DON'T KNOW/NO ANSWER"))

Did you give away any part of your AFRIPAD pack? Would you say...[READ]*

( ) Yes, the whole thing?
( ) Yes, part of the pack? Or
( ) No, none of it?
( ) [DO NOT READ] DON'T KNOW/NO ANSWER

Logic: Hidden unless: (Question "Did you sell any part of your AFRIPAD pack? Would you say...[READ]" #121 is one of the following answers ("Yes, part of the pack? Or") AND Question "Did you share any part of your AFRIPAD pack?" #123 is one of the following answers ("No","DON'T KNOW/NO ANSWER"))

Did you give away any part of your AFRIPAD pack? Would you say...[READ]*
( ) Yes, part of the pack? Or
( ) No, none of it?
( ) [DO NOT READ] DON'T KNOW/NO ANSWER

Logic: Hidden unless: (Question "Did you sell any part of your AFRIPAD pack? Would you say...[READ]" #122 is one of the following answers ("Yes, part of the pack? Or","No, none of it?","[DO NOT READ] DON'T KNOW/NO ANSWER") AND Question "Did you share any part of your AFRIPAD pack?" #123 is one of the following answers ("No","DON'T KNOW/NO ANSWER"))

Did you give away any part of your AFRIPAD pack? Would you say...[READ]*
( ) Yes, part of the pack? Or
( ) No, none of it?
( ) [DO NOT READ] DON'T KNOW/NO ANSWER

Logic: Hidden unless: ((Question "Did you give away any part of your AFRIPAD pack? Would you say...[READ]" #125 is one of the following answers ("Yes, the whole thing?","Yes, part of the pack? Or") OR Question "Did you give away any part of your AFRIPAD pack? Would you say...[READ]" #126 is one of the following answers ("Yes, part of the pack? Or")) OR Question "Did you give away any part of your AFRIPAD pack? Would you say...[READ]" #127 is one of the following answers ("Yes, part of the pack? Or"))

Who did you give it to?
[CHECK ALL THAT APPLY]*
[ ] Family
[ ] Friends
[ ] Other girls at school
[ ] Other (specify): ____*
[ ] DON'T KNOW/NO ANSWER

Logic: Hidden unless: (Question "Since the beginning of this school year, what do you USUALLY use to catch/absorb your MP? [*This question will enable more questions below.*] [IF GIRL STATES MORE THAN ONE ASK, WHAT DO YOU MOST FREQUENTLY USE?]" #45 is one of the following answers ("AFRIPAD") OR Question "Did you...[READ]" #116 is one of the following answers ("Use them only once?","Use them more than once and are still using them? Or","Use them more than once but stop using them?"))

How much did you like or dislike the AFRIPAD? Did you...[READ]*
( ) Like it very much
( ) Like it a little
( ) Dislike it a little, or
( ) Dislike it very much?
( ) [IF VOLUNTEERED] Neither
( ) [DO NOT READ] DON'T KNOW/NO ANSWER

Logic: Hidden unless: (Question "Since the beginning of this school year, what do you USUALLY use to catch/absorb your MP? [*This question will enable more questions below.*] [IF GIRL STATES MORE THAN ONE ASK, WHAT DO YOU MOST FREQUENTLY USE?]" #45 is one of the following answers ("AFRIPAD") OR Question "Did you...[READ]" #116 is one of the following answers ("Use them more than once and are still using them? Or","Use them more than once but stop using them?"))

During the day, how many hours did you usually wear the AFRIPAD before you had to change it?*

[ENTER NUMBER] [IF GIRL DOES NOT KNOW, ENTER DK] [IF NO ANSWER, ENTER 999]

____

Logic: Hidden unless: (Question "Since the beginning of this school year, what do you USUALLY use to catch/absorb your MP? [*This question will enable more questions below.*] [IF GIRL STATES MORE THAN ONE ASK, WHAT DO YOU MOST FREQUENTLY USE?]" #45 is one of the following answers ("AFRIPAD") OR Question "Did you...[READ]" #116 is one of the following answers ("Use them more than once and are still using them? Or","Use them more than once but stop using them?"))

Did you ever wear it for longer than 8 hours? Would you say...[READ]*

( ) Yes, usually
( ) Yes, but only on light days or at night, or
( ) No, never?
( ) [DO NOT READ] DON'T KNOW/NO ANSWER

Logic: Show/hide trigger exists. Hidden unless: (Question "Since the beginning of this school year, what do you USUALLY use to catch/absorb your MP? [*This question will enable more questions below.*] [IF GIRL STATES MORE THAN ONE ASK, WHAT DO YOU MOST FREQUENTLY USE?]" #45 is one of the following answers ("AFRIPAD") OR Question "Did you...[READ]" #116 is one of the following answers ("Use them more than once and are still using them? Or","Use them more than once but stop using them?"))

Were you able to go to school for a full day with only one AFRIPAD? Would you say...[READ]*

( ) Yes, always
( ) Yes, on some days, but not others, depending on the flow, or
( ) No, never?
( ) [DO NOT READ] DON'T KNOW/NO ANSWER

Logic: Hidden unless: Question "Were you able to go to school for a full day with only one AFRIPAD? Would you say...[READ]" #132 is one of the following answers ("Yes, on some days, but not others, depending on the flow, or","No, never?")

How much of a problem is it to change your AFRIPAD at school? Is it...[READ]*

( ) Not a problem at all,
( ) A little bit of a problem, or
( ) A big problem?
( ) [DO NOT READ] DON'T KNOW/NO ANSWER

Logic: Hidden unless: (Question "Did you...[READ]" #116 is one of the following answers ("Use them only once?","Use them more than once and are still using them? Or","Use them more than once but stop using them?") OR Question "Since the beginning of this school year, what do you USUALLY use to catch/absorb your MP? [*This question will enable more questions below.*]

[IF GIRL STATES MORE THAN ONE ASK, WHAT DO YOU MOST FREQUENTLY USE?]" #45 is one of the following answers ("AFRIPAD"))

How much of a problem is leaking while using an AFRIPAD? Is it...[READ]*
( ) Not a problem at all,
( ) A little bit of a problem, or
( ) A big problem?
( ) [DO NOT READ] DON'T KNOW/NO ANSWER

Logic: Hidden unless: (Question "Did you...[READ]" #116 is one of the following answers ("Use them more than once and are still using them? Or") OR Question "Since the beginning of this school year, what do you USUALLY use to catch/absorb your MP? [*This question will enable more questions below.*]
[IF GIRL STATES MORE THAN ONE ASK, WHAT DO YOU MOST FREQUENTLY USE?]" #45 is one of the following answers ("AFRIPAD"))

[AFRIPAD] When you're on your MP, do you think you can rely on this method to keep you from soiling when you have to:
Walk a long distance*
( ) Yes
( ) No
( ) NOT APPLICABLE
( ) DON'T KNOW/NO ANSWER

Logic: Hidden unless: (Question "Did you...[READ]" #116 is one of the following answers ("Use them more than once and are still using them? Or") OR Question "Since the beginning of this school year, what do you USUALLY use to catch/absorb your MP? [*This question will enable more questions below.*]
[IF GIRL STATES MORE THAN ONE ASK, WHAT DO YOU MOST FREQUENTLY USE?]" #45 is one of the following answers ("AFRIPAD"))

[AFRIPAD] When you're on your MP, do you think you can rely on this method to keep you from soiling when you have to:
Be gone from home for a long time*
( ) Yes
( ) No
( ) NOT APPLICABLE
( ) DON'T KNOW/NO ANSWER

Logic: Hidden unless: (Question "Did you...[READ]" #116 is one of the following answers ("Use them more than once and are still using them? Or") OR Question "Since the beginning of this school year, what do you USUALLY use to catch/absorb your MP? [*This question will enable more questions below.*]
[IF GIRL STATES MORE THAN ONE ASK, WHAT DO YOU MOST FREQUENTLY USE?]" #45 is one of the following answers ("AFRIPAD"))

[AFRIPAD] When you're on your MP, do you think you can rely on this method to keep you from soiling when you have to:
Move quickly or strenuously*
( ) Yes
( ) No
( ) NOT APPLICABLE
( ) DON'T KNOW/NO ANSWER

Logic: Hidden unless: (Question "Did you...[READ]" #116 is one of the following answers ("Use them more than once and are still using them? Or") OR Question "Since the beginning of this

school year, what do you USUALLY use to catch/absorb your MP? [*This question will enable more questions below.*]
[IF GIRL STATES MORE THAN ONE ASK, WHAT DO YOU MOST FREQUENTLY USE?]" #45 is one of the following answers ("AFRIPAD"))

[AFRIPAD] When you're on your MP, do you think you can rely on this method to keep you from soiling when you have to:
Sit for a long time*
( ) Yes
( ) No
( ) NOT APPLICABLE
( ) DON'T KNOW/NO ANSWER

Logic: Hidden unless: (Question "Did you...[READ]" #116 is one of the following answers ("Use them more than once and are still using them? Or") OR Question "Since the beginning of this school year, what do you USUALLY use to catch/absorb your MP? [*This question will enable more questions below.*]
[IF GIRL STATES MORE THAN ONE ASK, WHAT DO YOU MOST FREQUENTLY USE?]" #45 is one of the following answers ("AFRIPAD"))

[AFRIPAD] When you're on your MP, do you think you can rely on this method to keep you from soiling when you have to:
Be around males*
( ) Yes
( ) No
( ) NOT APPLICABLE
( ) DON'T KNOW/NO ANSWER

Logic: Hidden unless: (Question "Did you...[READ]" #116 is one of the following answers ("Use them more than once and are still using them? Or") OR Question "Since the beginning of this school year, what do you USUALLY use to catch/absorb your MP? [*This question will enable more questions below.*]
[IF GIRL STATES MORE THAN ONE ASK, WHAT DO YOU MOST FREQUENTLY USE?]" #45 is one of the following answers ("AFRIPAD"))

[AFRIPAD] When you're on your MP, do you think you can rely on this method to keep you from soiling when you have to:
Go to the farm away from home*
( ) Yes
( ) No
( ) NOT APPLICABLE
( ) DON'T KNOW/NO ANSWER

Logic: Hidden unless: (Question "Did you...[READ]" #116 is one of the following answers ("Use them more than once and are still using them? Or") OR Question "Since the beginning of this school year, what do you USUALLY use to catch/absorb your MP? [*This question will enable more questions below.*]
[IF GIRL STATES MORE THAN ONE ASK, WHAT DO YOU MOST FREQUENTLY USE?]" #45 is one of the following answers ("AFRIPAD"))

[AFRIPAD] When you're on your MP, do you think you can rely on this method to keep you from soiling when you have to:
Go to school*
( ) Yes
( ) No
( ) NOT APPLICABLE

( ) DON'T KNOW/NO ANSWER

**Logic: Hidden unless: (Question "Did you...[READ]" #116 is one of the following answers ("Use them more than once and are still using them? Or") OR Question "Since the beginning of this school year, what do you USUALLY use to catch/absorb your MP? [*This question will enable more questions below.*]
[IF GIRL STATES MORE THAN ONE ASK, WHAT DO YOU MOST FREQUENTLY USE?]" #45 is one of the following answers ("AFRIPAD"))**

[AFRIPAD] When you're on your MP, do you think you can rely on this method to keep you from soiling when you have to:
Go to the market*
( ) Yes
( ) No
( ) NOT APPLICABLE
( ) DON'T KNOW/NO ANSWER

**Logic: Show/hide trigger exists. Hidden unless: (Question "Did you...[READ]" #116 is one of the following answers ("Use them more than once and are still using them? Or","Use them more than once but stop using them?") OR Question "Since the beginning of this school year, what do you USUALLY use to catch/absorb your MP? [*This question will enable more questions below.*]
[IF GIRL STATES MORE THAN ONE ASK, WHAT DO YOU MOST FREQUENTLY USE?]" #45 is one of the following answers ("AFRIPAD"))**

Did you ever wash the AFRIPAD?*
( ) Yes (trigger)
( ) No (trigger)
( ) DON'T KNOW/NO ANSWER

Logic: Hidden unless: Question "Did you ever wash the AFRIPAD?" #143 is one of the following answers ("No (trigger)")

Why didn't you try to wash the AFRIPAD?
[CHECK ALL THAT APPLY]*
[ ] Had trouble getting water
[ ] Could not afford soap
[ ] Could afford soap but just didn't have any
[ ] Did not have enough time
[ ] Decided not to use it again
[ ] Did not have enough privacy
[ ] It was disgusting
[ ] Other (specify): ____*
[ ] DON'T KNOW/NO ANSWER

Logic: Show/hide trigger exists. Hidden unless: Question "Did you ever wash the AFRIPAD?" #143 is one of the following answers ("Yes (trigger)")

Did you use soap...[READ]*
( ) Every time,
( ) Sometimes, or
( ) Never?
( ) [DO NOT READ] DON'T KNOW/NO ANSWER

Logic: Hidden unless: Question "Did you use soap...[READ]" #145 is one of the following answers ("Sometimes, or","Never?")

Why didn't you use soap?
[CHECK ALL THAT APPLY]*

[ ] Often don't have soap
[ ] Don't need soap to get it clean
[ ] Don't care to use soap
[ ] Other (specify): ____*
[ ] DON'T KNOW/NO ANSWER

Logic: Hidden unless: Question "Did you ever wash the AFRIPAD?" #143 is one of the following answers ("Yes (trigger)")

Did you use hot water or cold water?*

( ) Hot water
( ) Cold water
( ) DON'T KNOW/NO ANSWER

Logic: Hidden unless: Question "Did you ever wash the AFRIPAD?" #143 is one of the following answers ("Yes (trigger)")

Was the water clean or dirty?*

( ) Clean
( ) Dirty
( ) DON'T KNOW/NO ANSWER

Logic: Hidden unless: Question "Did you ever wash the AFRIPAD?" #143 is one of the following answers ("Yes (trigger)")

Did you feel disgusted by washing the pad?*

( ) Yes
( ) No
( ) DON'T KNOW/NO ANSWER

Logic: Hidden unless: Question "Did you ever wash the AFRIPAD?" #143 is one of the following answers ("Yes (trigger)")

Did you worry about being observed washing the pad?*

( ) Yes
( ) No
( ) DON'T KNOW/NO ANSWER

Logic: Show/hide trigger exists. Hidden unless: Question "Did you ever wash the AFRIPAD?" #143 is one of the following answers ("Yes (trigger)")

Did you try to dry the pad?*

( ) Yes (trigger)
( ) No (trigger)
( ) DON'T KNOW/NO ANSWER

Logic: Hidden unless: Question "Did you try to dry the pad?" #151 is one of the following answers ("No (trigger)")

Why didn't you try to dry the pad?
[CHECK ALL THAT APPLY]*

[ ] Did not have enough time
[ ] Decided not to use it again
[ ] Did not have enough privacy
[ ] It was disgusting
[ ] Other (specify): ____*
[ ] DON'T KNOW/NO ANSWER

Logic: Hidden unless: Question "Did you try to dry the pad?" #151 is one of the following answers ("Yes (trigger)")

Where did you hang the pad to dry?
[CHECK ALL THAT APPLY]*
[ ] Dried pad under bed
[ ] Dried pad outdoors
[ ] Dried pad in dorm where other girls could see, such as on a peg
[ ] Dried pad in another secret place
[ ] Other (specify): ____*
[ ] DON'T KNOW/NO ANSWER

Logic: Hidden unless: Question "Did you try to dry the pad?" #151 is one of the following answers ("Yes (trigger)")

How many hours did it take to dry the pad completely?*
[ENTER NUMBER] [IF GIRL DOES NOT KNOW, ENTER DK] [IF NO ANSWER, ENTER 999]
____

Logic: Hidden unless: Question "Did you try to dry the pad?" #151 is one of the following answers ("Yes (trigger)")

Did you worry about drying the pad?*
( ) Yes
( ) No
( ) DON'T KNOW/NO ANSWER

Logic: Hidden unless: Question "Did you ever wash the AFRIPAD?" #143 is one of the following answers ("Yes (trigger)")

How often did you wear the pad damp? Would you say...[READ]*
( ) Usually
( ) Sometimes, or
( ) Never?
( ) [DO NOT READ] DON'T KNOW/NO ANSWER

Logic: Show/hide trigger exists. Hidden unless: (Question "Did you...[READ]" #116 is one of the following answers ("Use them more than once and are still using them? Or","Use them more than once but stop using them?") OR Question "Since the beginning of this school year, what do you USUALLY use to catch/absorb your MP? [*This question will enable more questions below.*]
[IF GIRL STATES MORE THAN ONE ASK, WHAT DO YOU MOST FREQUENTLY USE?]" #45 is one of the following answers ("AFRIPAD"))

If cost was not an issue, would you choose AFRIPADS over other methods of sanitary care?*
( ) Yes, would choose AFRIPADs
( ) No, would choose other methods
( ) DON'T KNOW/NO ANSWER

Logic: Hidden unless: Question "If cost was not an issue, would you choose AFRIPADS over other methods of sanitary care?" #157 is one of the following answers ("No, would choose other methods")

What would you choose instead of AFRIPADs?*
( ) Sanitary pads
( ) Cloth from home
( ) Another manufactured cloth pad
( ) Toilet roll
( ) Other (specify): ____*
( ) DON'T KNOW/NO ANSWER

**Logic: Hidden unless: (Question "Did you...[READ]" #116 is one of the following answers ("Use them more than once and are <u>still</u> using them? Or","Use them more than once but <u>stop</u> using them?") OR Question "Since the beginning of this school year, what do you USUALLY use to catch/absorb your MP? [*This question will enable more questions below.*]
[IF GIRL STATES MORE THAN ONE ASK, WHAT DO YOU MOST FREQUENTLY USE?]" #45 is one of the following answers ("AFRIPAD"))**

AFRIPADS cost 15 thousand shillings and last for about a year. Would you be able to afford them if you wanted to buy them?*
( ) Yes
( ) No
( ) Maybe
( ) DON'T KNOW/NO ANSWER

**Logic: Hidden unless: (Question "Did you...[READ]" #116 is one of the following answers ("Use them more than once and are <u>still</u> using them? Or","Use them more than once but <u>stop</u> using them?") OR Question "Since the beginning of this school year, what do you USUALLY use to catch/absorb your MP? [*This question will enable more questions below.*]
[IF GIRL STATES MORE THAN ONE ASK, WHAT DO YOU MOST FREQUENTLY USE?]" #45 is one of the following answers ("AFRIPAD"))**

If you had 15 thousand shillings, how likely would you be to buy the AFRIPADS?...[READ]*
( ) Not at all likely
( ) Slightly likely
( ) Moderately likely
( ) Very likely
( ) Extremely likely
( ) [DO NOT READ] DON'T KNOW/NO ANSWER

**Logic: Hidden unless: (Question "Since the beginning of this school year, what do you USUALLY use to catch/absorb your MP? [*This question will enable more questions below.*]
[IF GIRL STATES MORE THAN ONE ASK, WHAT DO YOU MOST FREQUENTLY USE?]" #45 is one of the following answers ("AFRIPAD") OR Question "Did you...[READ]" #116 is one of the following answers ("Use them only once?","Use them more than once and are <u>still</u> using them? Or","Use them more than once but <u>stop</u> using them?"))**

Do you have any suggestions for improving the AFRIPAD?*
( ) Yes (specify): ____*
( ) No
( ) DON'T KNOW/NO ANSWER

Logic: Hidden unless: Question "Since the beginning of this school year, what do you USUALLY use to catch/absorb your MP? [*This question will enable more questions below.*]

[IF GIRL STATES MORE THAN ONE ASK, WHAT DO YOU MOST FREQUENTLY USE?]" #45 is one of the following answers ("AFRIPAD")

Since we came last year, did you use any other sanitary products for your MP other than the AFRIPADs? If so, what?
[CHECK ALL THAT APPLY]*
[ ] Bought sanitary pads from shops
[ ] Cloth
[ ] Other (specify): ____*
[ ] *I used nothing else.*
[ ] DON'T KNOW/NO ANSWER

Menstrual Practices

Logic: Show/hide trigger exists.

Page 7 - [RA: INTERVIEW DISPOSITION]*
( ) Consent obtained and MP
( ) Consent obtained but NO MP
( ) No consent

Logic: Hidden unless: Question "Page 7 - [RA: INTERVIEW DISPOSITION]" is one of the following answers ("Consent obtained and MP")

Now, I'm going to ask you some more questions about your MP.

Logic: Show/hide trigger exists. Hidden unless: Question "Page 7 - [RA: INTERVIEW DISPOSITION]" is one of the following answers ("Consent obtained and MP")

How frequently do you change your sanitary protection (either pad/cloth/toilet roll/other?)
[RA: DAY = 24 hrs]*
( ) 1 time a day
( ) 2 times a day
( ) 3 times a day
( ) 4 times a day
( ) Over 5 times a day
( ) Never change protection
( ) DON'T KNOW/NO ANSWER

Logic: Show/hide trigger exists. Hidden unless: Question "How frequently do you change your sanitary protection (either pad/cloth/toilet roll/other?)
[RA: DAY = 24 hrs]" #163 is one of the following answers ("1 time a day","2 times a day","3 times a day","4 times a day","Over 5 times a day","DON'T KNOW/NO ANSWER")

When you are not at home or school and need to change your sanitary protection, what do you usually do? Do you...[READ]*
( ) Immediately go home to change?
( ) Wait until you are back home to change? Or
( ) Change while away from home?
( ) [DO NOT READ]DON'T KNOW/NO ANSWER

Logic: Hidden unless: Question "When you are not at home or school and need to change your sanitary protection, what do you usually do? Do you...[READ]" #164 is one of the following answers ("Immediately go home to change?","Wait until you are back home to change? Or")

Why do you change at home?
[CHECK ALL THAT APPLY]*
[ ] Do not carry extra sanitary protection with me
[ ] Do not have enough privacy
[ ] Do not know where to dispose of sanitary protection away from home
[ ] Other (specify): ____*
[ ] DON'T KNOW/NO ANSWER

Logic: Hidden unless: Question "When you are not at home or school and need to change your sanitary protection, what do you usually do? Do you...[READ]" #164 is one of the following answers ("Change while away from home?")

What do you USUALLY do with your used sanitary protection when you have to change away from home or school?
[IF GIRL STATES MORE THAN ONE ASK, WHAT DO YOU MOST FREQUENTLY DO WITH YOUR USED SANITARY PROTECTION AWAY FROM HOME OR SCHOOL?]*
( ) Throw in latrine
( ) Throw in bush
( ) Carry back home and wash and reuse
( ) Carry back home and dispose there
( ) Dispose at community rubbish heap
( ) Bury
( ) Other (specify): ____*
( ) DON'T KNOW/NO ANSWER

Logic: Hidden unless: Question "Page 7 - [RA: INTERVIEW DISPOSITION]" is one of the following answers ("Consent obtained and MP")

How many minutes does it usually take for you to change your sanitary protection?*
[ENTER NUMBER OF MINUTES] [IF GIRL DOESN'T KNOW, INPUT DK] [IF NO ANSWER, INPUT 999]

____

Logic: Hidden unless: Question "Page 7 - [RA: INTERVIEW DISPOSITION]" is one of the following answers ("Consent obtained and MP")

Do you bathe more often when you are on your MP?*
( ) Yes
( ) No
( ) DON'T KNOW/NO ANSWER

Logic: Hidden unless: Question "Page 7 - [RA: INTERVIEW DISPOSITION]" is one of the following answers ("Consent obtained and MP")

Do you have regular access to soap at home?*
( ) Yes
( ) No
( ) DON'T KNOW/NO ANSWER

Logic: Hidden unless: Question "Page 7 - [RA: INTERVIEW DISPOSITION]" is one of the following answers ("Consent obtained and MP")

Do you have regular access to water at home?*
( ) Yes
( ) No
( ) DON'T KNOW/NO ANSWER

Logic: Hidden unless: Question "Page 7 - [RA: INTERVIEW DISPOSITION]" is one of the following answers ("Consent obtained and MP")

Do you have regular access to soap at school?*
( ) Yes
( ) No
( ) DON'T KNOW/NO ANSWER

Logic: Hidden unless: Question "Page 7 - [RA: INTERVIEW DISPOSITION]" is one of the following answers ("Consent obtained and MP")

Do you have regular access to water at school?*
( ) Yes
( ) No
( ) DON'T KNOW/NO ANSWER

Logic: Show/hide trigger exists. Hidden unless: Question "Page 7 - [RA: INTERVIEW DISPOSITION]" is one of the following answers ("Consent obtained and MP")

Do you have underwear?*
( ) Yes (trigger)
( ) No
( ) DON'T KNOW/NO ANSWER

Logic: Hidden unless: Question "Do you have underwear?" #173 is one of the following answers ("Yes (trigger)")

Do you usually wear underwear with your sanitary protection?*
( ) Yes
( ) No
( ) DON'T KNOW/NO ANSWER

Logic: Show/hide trigger exists. Hidden unless: Question "Page 7 - [RA: INTERVIEW DISPOSITION]" is one of the following answers ("Consent obtained and MP")

Are you able to go to school for a full day without changing your sanitary protection? Would you say...[READ]*
( ) Yes, always
( ) Yes, on some days, but not others, depending on the flow, or
( ) No, never?
( ) [DO NOT READ] DON'T KNOW/NO ANSWER

Logic: Hidden unless: Question "Are you able to go to school for a full day without changing your sanitary protection? Would you say...[READ]" #175 is one of the following answers ("Yes, on some days, but not others, depending on the flow, or","No, never?")

How much of a problem is it to change your sanitary protection at school? Is it...[READ]*
( ) Not a problem at all
( ) A little bit of a problem, or
( ) A big problem?
( ) [DO NOT READ] DON'T KNOW/NO ANSWER

Logic: Hidden unless: Question "Are you able to go to school for a full day without changing your sanitary protection? Would you say...[READ]" #175 is one of the following answers ("Yes, on some days, but not others, depending on the flow, or","No, never?")

What do you do when you need to change your sanitary protection at school?*
( ) Ask permission from teacher
( ) Leave without permission
( ) Sit and wait until others are gone
( ) Other (specify): ____*
( ) DON'T KNOW/NO ANSWER

Logic: Hidden unless: Question "Are you able to go to school for a full day without changing your sanitary protection? Would you say...[READ]" #175 is one of the following answers ("Yes, on some days, but not others, depending on the flow, or","No, never?")

What do you USUALLY do with your used sanitary protection when you have to change <u>at school</u>?
[IF GIRL STATES MORE THAN ONE ASK, WHAT DO YOU MOST FREQUENTLY DO WITH YOUR USED SANITARY PROTECTION AT SCHOOL?]*
( ) Throw in latrine
( ) Throw in bush
( ) Carry back home and wash and reuse
( ) Carry back home and dispose there
( ) Dispose at community rubbish heap
( ) Bury
( ) Go home to change
( ) Don't go to school if need to change
( ) Other (specify): ____*
( ) DON'T KNOW/NO ANSWER

Logic: Hidden unless: Question "Page 7 - [RA: INTERVIEW DISPOSITION]" is one of the following answers ("Consent obtained and MP")

What do you USUALLY do with your used sanitary protection when you change <u>at home</u>?
[IF GIRL STATES MORE THAN ONE ASK, WHAT DO YOU MOST FREQUENTLY DO WITH YOUR USED SANITARY PROTECTION AT HOME?]*
( ) Throw in latrine
( ) Throw in bush
( ) Put in trash at home
( ) Dispose at community rubbish heap
( ) Wash at home and reuse
( ) Bury
( ) Burn
( ) Other (specify): ____*
( ) DON'T KNOW/NO ANSWER

Health, School, and Activities

Logic: Show/hide trigger exists.

Page 8 - [RA: INTERVIEW DISPOSITION]*
( ) Consent obtained and MP
( ) Consent obtained but <u>NO MP</u>
( ) No consent

Logic: Hidden unless: Question "Page 8 - [RA: INTERVIEW DISPOSITION]" is one of the following answers ("Consent obtained and MP")

Have you ever used a sanitary pad?*
( ) Yes
( ) No
( ) Not Applicable
( ) DON'T KNOW/NO ANSWER

Logic: Show/hide trigger exists. Hidden unless: Question "Page 8 - [RA: INTERVIEW DISPOSITION]" is one of the following answers ("Consent obtained and MP")

Since the start of the school year, have you ever experienced any itching or burning in the pelvic area? This could be when you are on your MP or at other times during the month.*
( ) Yes (trigger)
( ) No
( ) DON'T KNOW/NO ANSWER

Logic: Hidden unless: Question "Since the start of the school year, have you ever experienced any itching or burning in the pelvic area? This could be when you are on your MP or at other times during the month." #181 is one of the following answers ("Yes (trigger)")

Did you experience the itching or burning while on your MP? At other times? Or both?*
( ) While on MP
( ) At other times
( ) Both
( ) DON'T KNOW/NO ANSWER

Logic: Show/hide trigger exists. Hidden unless: Question "Page 8 - [RA: INTERVIEW DISPOSITION]" is one of the following answers ("Consent obtained and MP")

Since the start of the school year, have you ever had any white or green discharge from your vagina?*
( ) Yes (trigger)
( ) No
( ) DON'T KNOW/NO ANSWER

Logic: Hidden unless: Question "Since the start of the school year, have you ever had any white or green discharge from your vagina?" #183 is one of the following answers ("Yes (trigger)")

Did you experience the discharge while on your MP? At other times? Or both?*
( ) While on MP
( ) At other times
( ) Both
( ) DON'T KNOW/NO ANSWER

Logic: Show/hide trigger exists. Hidden unless: Question "Page 8 - [RA: INTERVIEW DISPOSITION]" is one of the following answers ("Consent obtained and MP")

How often do you worry about odor during your MP? Would you say...[READ]*
( ) Never,
( ) Rarely,
( ) Sometimes,
( ) Often, or
( ) All the time?

( ) [DO NOT READ] DON'T KNOW/NO ANSWER

Logic: Hidden unless: Question "How often do you worry about odor during your MP? Would you say...[READ]" #185 is one of the following answers ("Rarely,","Sometimes,","Often, or","All the time?")

In what situations do you worry about odor?
[CHECK ALL THAT APPLY]*

[ ] At school
[ ] Around males
[ ] In other social environments (on the bus, at church, at the market, etc.)
[ ] Other (specify): ____*
[ ] DON'T KNOW/NO ANSWER

Logic: Show/hide trigger exists. Hidden unless: Question "Page 8 - [RA: INTERVIEW DISPOSITION]" is one of the following answers ("Consent obtained and MP")

Does your MP ever cause you to...[READ]
[CHECK ALL THAT APPLY]*

[ ] Miss school
[ ] Not do your homework
[ ] Miss work in the field/gardens
[ ] Be unable to play with other children
[ ] Avoid physical sports/exercise
[ ] Stay indoors
[ ] Avoid being around males
[ ] Avoid chores
[ ] Avoid sex
[ ] Anything else? (specify): ____*
[ ] [DO NOT READ] DON'T KNOW/NO ANSWER

Logic: Hidden unless: Question "Does your MP ever cause you to...[READ] [CHECK ALL THAT APPLY]" #187 is one of the following answers ("Miss school")

How many days of school do you miss during your MP?*
[ENTER NUMBER] [IF GIRL DOESN'T KNOW, ENTER DK] [IF NO ANSWER, ENTER 999]

____

Logic: Hidden unless: Question "Does your MP ever cause you to...[READ] [CHECK ALL THAT APPLY]" #187 is one of the following answers ("Miss school")

Why did you miss school?
[CHECK ALL THAT APPLY]*

[ ] Cramps/bad physical feelings
[ ] Fear of leaking
[ ] Don't have (lost)/can't afford pads
[ ] Other (specify): ____*
[ ] DON'T KNOW/NO ANSWER

Logic: Hidden unless: Question "Does your MP ever cause you to...[READ] [CHECK ALL THAT APPLY]" #187 is one of the following answers ("Miss school")

Which of the following causes you to miss school the most?...[READ]*

( ) Physical pain,
( ) Fear of soiling, or
( ) Not having any sanitary protection?

( ) [DO NOT READ] DON'T KNOW/NO ANSWER

Logic: Hidden unless: Question "Page 8 - [RA: INTERVIEW DISPOSITION]" is one of the following answers ("Consent obtained and MP")

What reasons besides your MP cause you to miss school? [READ IF NECESSARY] [CHECK ALL THAT APPLY]*
[ ] Being ill for other reasons
[ ] Household duties
[ ] Helping on the farm
[ ] Tending to others who are sick
[ ] Bad weather
[ ] Earning money for the household
[ ] Too far
[ ] No uniform
[ ] No supplies
[ ] Caring for smaller children
[ ] Lack of school dues
[ ] Problems with children at school teasing/bullying
[ ] Problems with teachers at school
[ ] Any other reasons? (specify): ____*
[ ] [DO NOT READ] DON'T KNOW/NO ANSWER

Logic: Show/hide trigger exists. Hidden unless: Question "Page 8 - [RA: INTERVIEW DISPOSITION]" is one of the following answers ("Consent obtained and MP")

How many minutes does it take you to get to school?*
( ) [ENTER MINUTES]: ____*
( ) Boards at school
( ) DON'T KNOW/NO ANSWER

Logic: Hidden unless: Question "How many minutes does it take you to get to school?" #192 is one of the following answers ("[ENTER MINUTES]","DON'T KNOW/NO ANSWER")

How do you usually get to school?*
( ) Walk
( ) Ride the bus
( ) Other (specify): ____*
( ) DON'T KNOW/NO ANSWER

Logic: Show/hide trigger exists. Hidden unless: Question "Page 8 - [RA: INTERVIEW DISPOSITION]" is one of the following answers ("Consent obtained and MP")

Do you find it difficult to concentrate at school when you have your MP?*
( ) Yes (trigger)
( ) No
( ) DON'T KNOW/NO ANSWER

Logic: Hidden unless: Question "Do you find it difficult to concentrate at school when you have your MP?" #194 is one of the following answers ("Yes (trigger)")

Why? [READ IF NECESSARY]
[CHECK ALL THAT APPLY]*
[ ] Actual soiling
[ ] Fear of soiling
[ ] Scent

[ ] Discomfort
[ ] Actual teasing
[ ] Fear of teasing
[ ] Cramps
[ ] Any other reasons? (specify): ____*
[ ] [DO NOT READ] DON'T KNOW/NO ANSWER

Logic: Hidden unless: Question "Page 8 - [RA: INTERVIEW DISPOSITION]" is one of the following answers ("Consent obtained and MP")

Do you avoid standing in class to answer questions during your MP?*
( ) Yes
( ) No
( ) DON'T KNOW/NO ANSWER

Logic: Show/hide trigger exists. Hidden unless: Question "Page 8 - [RA: INTERVIEW DISPOSITION]" is one of the following answers ("Consent obtained and MP")

Are there activities that are forbidden to you while you are on your MP?*
( ) Yes (trigger)
( ) No
( ) DON'T KNOW/NO ANSWER

Logic: Hidden unless: Question "Are there activities that are forbidden to you while you are on your MP?" #197 is one of the following answers ("Yes (trigger)")

What activities are forbidden to you while you are on your MP?
[CHECK ALL THAT APPLY]*
[ ] Serving food and beverages to guests
[ ] Fetching water
[ ] Serving food and beverages to men
[ ] Cooking
[ ] Being in a sacred space
[ ] Other (specify): ____*
[ ] DON'T KNOW/NO ANSWER

Logic: Show/hide trigger exists. Hidden unless: Question "Page 8 - [RA: INTERVIEW DISPOSITION]" is one of the following answers ("Consent obtained and MP")

Are there activities or settings you avoid while you are on your MP?*
( ) Yes (trigger)
( ) No
( ) DON'T KNOW/NO ANSWER

Logic: Hidden unless: Question "Are there activities or settings you avoid while you are on your MP?" #199 is one of the following answers ("Yes (trigger)")

What activities or settings do you avoid while you are on your MP?
[CHECK ALL THAT APPLY]*
[ ] Serving food and beverages to guests
[ ] Being around males
[ ] Fetching water
[ ] Cooking
[ ] Being in a sacred space
[ ] Physical sports/exercise
[ ] Playing with other children

[ ] Working in the field/garden
[ ] Going to school
[ ] Doing homework
[ ] Other (specify): ____*
[ ] DON'T KNOW/NO ANSWER

Menstrual Knowledge and Beliefs

Logic: Show/hide trigger exists.

Page 9 - [RA: INTERVIEW DISPOSITION]*
( ) Consent obtained and MP
( ) Consent obtained but NO MP
( ) No consent

Logic: Hidden unless: Question "Page 9 - [RA: INTERVIEW DISPOSITION]" is one of the following answers ("Consent obtained and MP")

During your MP, do you feel ashamed? Or do you feel about the same as when you are not on your MP?*
( ) Feel ashamed
( ) Feel about the same
( ) DON'T KNOW/NO ANSWER

Logic: Hidden unless: Question "Page 9 - [RA: INTERVIEW DISPOSITION]" is one of the following answers ("Consent obtained and MP")

Do you worry that other people can smell your MP? Or are you not worried about this?*
( ) Worry that others can smell MP
( ) Not worried about this
( ) DON'T KNOW/NO ANSWER

Logic: Hidden unless: Question "Page 9 - [RA: INTERVIEW DISPOSITION]" is one of the following answers ("Consent obtained and MP")

Do the boys you know tease girls about their MP?*
( ) Yes
( ) No
( ) DON'T KNOW/NO ANSWER

Logic: Hidden unless: Question "Page 9 - [RA: INTERVIEW DISPOSITION]" is one of the following answers ("Consent obtained and MP")

During your MP, do you feel insecure? Or do you feel about the same?*
( ) Feel insecure
( ) Feel about the same
( ) DON'T KNOW/NO ANSWER

Logic: Hidden unless: Question "Page 9 - [RA: INTERVIEW DISPOSITION]" is one of the following answers ("Consent obtained and MP")

Since you started your MP, do you ever worry about getting pregnant?*
( ) Yes
( ) No
( ) DON'T KNOW/NO ANSWER

Logic: Hidden unless: Question "Page 9 - [RA: INTERVIEW DISPOSITION]" is one of the following answers ("Consent obtained and MP")

Since you started your MP, does your family expect any of the following changes from you? [READ]
[CHECK ALL THAT APPLY]*

[ ] Finding a job
[ ] Leaving school
[ ] Getting married soon
[ ] [DO NOT READ] DON'T KNOW/NO ANSWER

Logic: Hidden unless: Question "Page 9 - [RA: INTERVIEW DISPOSITION]" is one of the following answers ("Consent obtained and MP")

Since you started your MP, does your family expect you to do more housework? Less housework? Or about the same amount of housework?*

( ) More housework
( ) Less housework
( ) About the same amount of housework
( ) DON'T KNOW/NO ANSWER

Logic: Hidden unless: Question "Page 9 - [RA: INTERVIEW DISPOSITION]" is one of the following answers ("Consent obtained and MP")

Since you started your MP, does your family give you more money? Less money? Or about the same amount of money?*

( ) More money
( ) Less money
( ) About the same amount of money
( ) My parents never gave me any money
( ) DON'T KNOW/NO ANSWER

Logic: Hidden unless: Question "Page 9 - [RA: INTERVIEW DISPOSITION]" is one of the following answers ("Consent obtained and MP")

Since you started your MP, do you feel more LIKE A WOMAN? Or do you feel about the same?*

( ) Feel more like a woman
( ) Feel about the same
( ) DON'T KNOW/NO ANSWER

Logic: Hidden unless: Question "Page 9 - [RA: INTERVIEW DISPOSITION]" is one of the following answers ("Consent obtained and MP")

Since you started your MP, do <u>others treat you</u> more LIKE A WOMAN? Or do they treat you the same?*

( ) Treat you more like a woman
( ) Treat you about the same
( ) DON'T KNOW/NO ANSWER

Admin

[RA: ENTER YOUR NAME]*

[ENTER INTERVIEW DATE (DD/MM/YYYY)]*

[RA DO YOU HAVE ANY COMMENTS YOU WOULD LIKE TO ADD FOR THIS GIRL'S RESPONSES OR OTHER CONDITIONS RELATED TO THIS GIRL]

____

Thank You!

Thank you for taking our survey. Your response is very important to us.
